# Intramolecular hydroamination catalysed by gold nanoparticles deposited on fibrillated cellulose

**DOI:** 10.1038/s41598-022-24955-3

**Published:** 2022-11-29

**Authors:** Yuta Uetake, Butsaratip Suwattananuruk, Hidehiro Sakurai

**Affiliations:** 1 Division of Applied Chemistry, Graduate School of Engineering, Osaka University, 2-1 Yamadaoka, Suita, Osaka 565-0871 Japan; 2 Innovative Catalysis Science Division, Institute for Open and Transdisciplinary Research Initiatives (ICS-OTRI), Osaka University, 2-1 Yamadaoka, Suita, Osaka 565-0871 Japan

**Keywords:** Catalysis, Other nanotechnology, Carbohydrate chemistry

## Abstract

Gold nanoparticles stabilised by fibrillated citric acid-modified cellulose (Au:F-CAC) catalyse the intramolecular cycloamination of amines to unactivated alkenes under an aerobic atmosphere to afford pyrrolidine derivatives. Only 0.2 mol% of Au loading is required to complete the reaction. The high sensitivity of the Au:F-CAC catalyst to the substitution pattern of alkenes allows a unique chemoselective cycloamination, affording new compounds.

## Introduction

**Figure 1:**
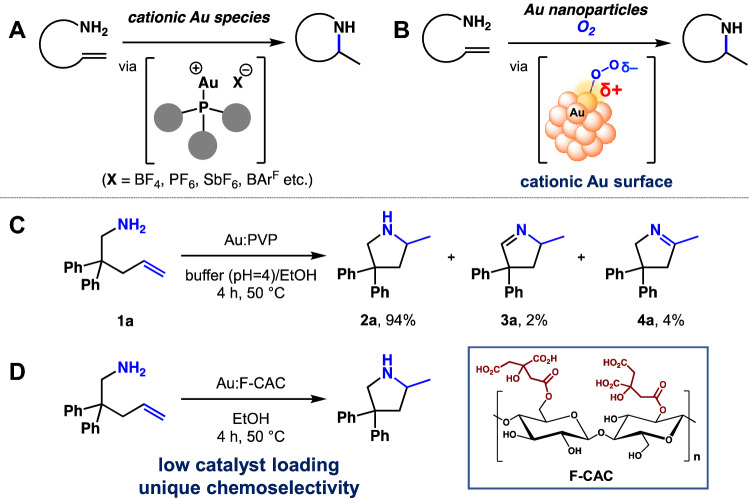
Cationic Au-catalysed intermolecular cycloamination reaction. (**A**) Homogeneous catalysis. (**B**) Heterogeneous catalysis. (**C**) Previous work using Au:PVP. (**D**) This work using Au:F-CAC. ArF: Tetrakis[3,5-bis(trifluoromethyl)phenyl].

The cationic gold complex is one of the most helpful metal catalysts to activate unsaturated bonds, such as alkenes and alkynes (Fig. 1A)^1–3^. The activated double or triple bonds react with nucleophiles both intermolecularly and intramolecularly to give functional or cyclic compounds^4,5^. The carbophilic and π-acidic nature of cationic gold species do not lose their electrophilicity in the presence of hard nucleophiles, such as oxygen or nitrogen, allowing the use of nucleophilic reagents, such as amines, hydroxy groups, and water^6,7^.

In the case of (quasi-)heterogeneous catalysis, gold nanoparticles (Au NPs) also show Lewis acidity in the presence of molecular oxygen. It is well known that super-oxide-like species, useful for catalytic aerobic oxidation reactions, are generated after the absorption of oxygen (Fig. 1B)^8^. At the same moment, the surface of gold near the absorption site becomes positively charged, resulting in Lewis acidity^9–11^. Previous studies revealed that intermolecular hydroamination^12,13^ proceeded in the presence of the Au:PVP [PVP: poly(*N*-vinyl-2-pyrrolidone)] under aerobic conditions (Fig. 1C)^14–16^. The terminal and/or internal alkenes were activated at the cationic site with the help of molecular oxygen to afford cyclic amines, commonly found in natural products and pharmaceutics. This study investigated other stabilising supports, mainly polymer-based matrices, which combine high catalytic activity with ease of use.

Our former studies proposed that Au NPs stabilised by biomacromolecules, such as chitin and chitosan, showed superior catalytic activity rather than that of PVP in some cases^17,18^. For example, Au NPs stabilised by chitin or chitosan were found suitable for homocoupling reaction of phenylboronic acids because of auxiliary functional groups, such as amine and amide^19^. The localised basic environment nearby the reaction site prevented the unfavoured oxidation pathway and therefore increased the chemoselectivity of this reaction. This example suggests that a functionalised polymer matrix can effectively control the response. Recently, we have developed a concise preparation method to fabricate fibrillated cellulose by chemical modification using citric acid (F-CAC)^20,21^. In addition, we have developed a size-selective preparation method to afford Au NPs stabilised on F-CAC (Au:F-CAC). They were applied for aerobic oxidation of benzylic alcohols^22,23^. Since the Au NP-catalysed hydroamination reaction proceeds under slightly acidic conditions (pH = 4), we considered that the F-CAC, bearing carboxylic acid moieties, is a suitable matrix to facilitate the hydroamination reaction. Additionally, the high tolerance against organic solvents and their recyclability renders the reaction practical. Herein, we report a matrix effect of F-CAC toward hydroamination reaction (Fig. 1D).

## Results and discussions

### Optimisation of reaction conditions

Our attempt commenced with an intramolecular hydroamination reaction of **1a** using Au:F-CAC (particle size of Au: 1.7 ± 0.2 nm, 1.68 × 10^–3^ wt%) prepared by the trans-deposition method^22,24,25^. In the presence of Au:F-CAC (5 mol%), the reaction was performed in buffer/EtOH solution under the aerobic condition to afford pyrrolidine **2a** in quantitative yield without any side products, such as **3a** or **4a** (Table 1, entry 1, and Table S1). It should be noted that intermolecular addition of ammonia did not proceed because of its low nucleophilicity, and entropically favorable intramolecular reaction occurs much faster. When the reaction was carried out without an additional formic acid, which acts as a reducing agent, the reaction did not proceed, indicating that the citric acid moiety in F-CAC does not work as a reducing agent (entry 2). Although a slight extension of the reaction time was required, the reaction took place with 2 mol% of Au:F-CAC without any loss of the yield of **2a** (entry 3). At this stage, we performed an inductively coupled plasma-atomic emission spectroscopy (ICP-AES) measurement of the Au:F-CAC after reaction to find severe leaching of gold from F-CAC (45% Au loss). As proposed in the previous work, the leaching of gold occurred when polar solvents, such as water, DMF, and DMSO, were used^22^. To reduce the amount of water in the solvent, we optimised the solvent system using 2 mol% of Au:F-CAC. The reaction did not proceed in toluene; however, we found that the use of absolute EtOH showed a better result (25% Au loss) than the case of the buffer/EtOH system (entry 4). Finally, the use of ammonium formate (500 mol%), instead of liquid ammonium and formic acid, in EtOH completely suppresses the leaching of gold with retaining its high catalytic activity (entry 5). Given our previous theoretical studies^26^, the use of excess amount of ammonium formate would accelerate the cleavage of Au–C bond to expel the cyclized product. Under the reaction conditions, the amount of gold could be reduced to 0.2 mol%, prolonging the reaction time by 8 h (entries 6 and 7). To compare their catalytic activities, we also performed the reaction using Au:PVP under the same conditions to find that Au:F-CAC showed a higher yield (85%) than Au:PVP (45%) at the point after 2 h (entries 8 and 9). The difference in catalytic activity was more pronounced when the amount of Au was compared at 0.2 mol% (entries 7 and 10), and the reaction did not complete even when the reaction was prolonged to 24 h in the case of Au:PVP (entry 11). These results suggested the high stability of Au NPs under the reaction conditions. Despite 0.2 mol% prolonging the reaction time of 8 h, the substrate scope is limited to **1a**. Thus, the reaction conditions of entry 6 were selected as an optimisation condition due to the requirement of an additional amount of gold in the substrates that have substituents at the alkene position (Fig. 3, vide infra).

**Table 1:** Optimisation of reaction conditions.


Entry	Au NPs (mol%)	Additives (mol%)	Time (h)	Recovery of **1a** (%)^a^	Yield of **2a** (%)^a^	Leaching (%)
1	Au:F-CAC (5)	HCO_2_H (1000) aq. NH_3_ (2000)	4	0	99	n.d.
2	Au:F-CAC (5)	aq. NH_3_ (500)	4	100	0	n.d.
3	Au:F-CAC (2)	HCO_2_H (1000) aq. NH_3_ (2000)	10	0	99	45
4^b^	Au:F-CAC (2)	HCO_2_H (1000) aq. NH_3_ (2000)	4	0	99	25
5^b^	Au:F-CAC (2)	HCO_2_NH_4_ (500)	4	0	99	0
6^b^	Au:F-CAC (0.5)	HCO_2_NH_4_ (500)	4	0	99 (99)^c^	n.d.
7^b^	Au:F-CAC (0.2)	HCO_2_NH_4_ (500)	8	0	99 (99)^c^	n.d.
8^b^	Au:F-CAC (0.5)	HCO_2_NH_4_ (500)	2	13	85	n.d.
9^b^	Au:PVP (0.5)	HCO_2_NH_4_ (500)	2	47	45	n.d.
10^b^	Au:PVP (0.2)	HCO_2_NH_4_ (500)	8	52	46	n.d.
11^b^	Au:PVP (0.2)	HCO_2_NH_4_ (500)	24	43	55	n.d.

^a^Determined by ^1^H NMR analysis.

^b^Absolute EtOH was used as a solvent.

^c^Isolated yield in parentheses.

### The durability and reusability of Au:F-CAC

To gain insight into the catalytic activity of Au:F-CAC, Au NPs after the reaction were observed by transmission electron microscopy (TEM). As in our previous investigation, the severe aggregation of Au:PVP was observed under the aqueous conditions (Table 2, entry C)^15^. Meanwhile, the aggregation of Au NPs was suppressed to some extent in the case of Au:F-CAC (entry A). The use of solid ammonium formate in absolute EtOH was found to suppress the aggregation in both cases. However, the particle size of Au:F-CAC was much smaller than that of Au:PVP (entries B and D), leading to the high catalytic activity of the Au:F-CAC catalyst.

**Table 2:** TEM images and particle sizes of Au:F-CAC and Au:PVP after reaction.


Entry	Catalyst^a^	Additive	Solvent	Particle size (nm)
A^b^	Au:F-CAC	HCO_2_H (1000) aq. NH_3_ (2000)	Buffer/EtOH	5.7 ± 1.8
B^c^	Au:F-CAC	HCO_2_NH_4_ (500)	EtOH	2.3 ± 0.7
C^d^	Au:PVP	HCO_2_H (1000) aq. NH_3_ (2000)	Buffer/EtOH	Aggregation
D^d^	Au:PVP	HCO_2_NH_4_ (500)	EtOH	3.2 ± 0.7

^a^2 mol% of Au NPs was used.

^b^Entry 4 in Table 1.

^c^Entry 5 in Table 1.

^d^Au:PVP(K-30) (1.5 ± 0.2 nm) was used.

**Figure 2:**
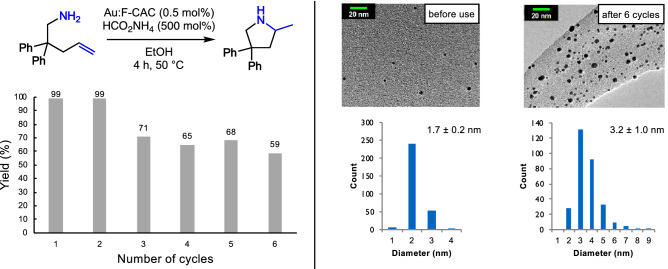
Reusability test of Au:F-CAC.

Next, the reusability test of Au:F-CAC catalyst was conducted to evaluate its durability (Fig. 2). After each reaction, the catalyst was filtered and washed with ethanol. After drying overnight, the spent catalyst was subjected to the next cycle. The hydroamination of **1a** proceeded almost quantitatively after 4 h in the 2nd cycle. Although a substantial decrease in the catalytic activity was observed after 3rd cycle, **2a** was still obtained in moderate yields (59–71%). TEM observations of the spent catalyst (after six cycles) showed slightly aggregated Au NPs (3.2 ± 0.2 nm), suggesting that the decrease of the catalytic activity would be ascribed to the decrease of the surface area of Au NPs.

### The effect of the reaction atmosphere

The atmosphere of the reaction was investigated using 0.5 mol% Au:F-CAC. The yield of **2a** was almost unchanged between aerobic and oxygen atmospheres, indicating that the partial pressure of oxygen does not affect the reaction rate (Table 3, entries 1 and 2). This result suggested that the absorption of oxygen atoms does not include in the rate-determining step of the reaction. Indeed, we reported a theoretical investigation of the reaction mechanism of hydroamination reaction using the density functional theory (DFT) method^26^, and concluded that the reductive elimination step would be the rate-determining step. Therefore, this experimental result is consistent with the theoretical calculations. As expected, the cyclisation did not proceed under a nitrogen atmosphere, indicating that oxygen is crucial for this reaction (entry 3).

**Table 3:** Effect of the reaction atmosphere.


Entry	Atmosphere	Time (h)	Recovery of **1a** (%)^a^	Yield of **2a** (%)^a^
1	Air	1	44	53
2	O_2_	1	43	56
3	N_2_	4	100	0

^a^Determined by ^1^H NMR analysis.

### Substrate scope

**Figure 3:**
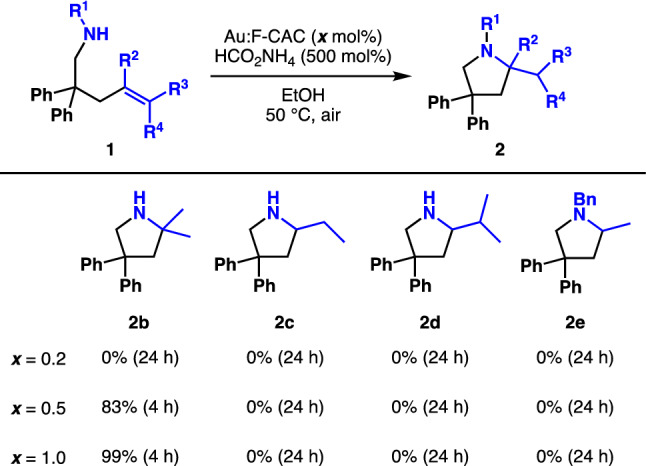
Scope of alkenes.

The substrates having different substituents and protecting groups were subjected to the reaction (Fig. 3). Although a primary amine having a β-methallyl group did not participate in this reaction under an optimised reaction condition (Table 1, entry 7), the cyclisation proceeded with 0.5 mol% Au loading to afford pyrrolidine **2b** in 83% yield. Moreover, **2b** was obtained quantitatively when 1.0 mol% Au:F-CAC was used. Primary amines bearing crotyl (**1c**) and prenyl group (**1d**) did not participate in the reaction even when extending time or increasing the amount of catalyst, probably because of sterically hindered di- or tri-substituted alkene. Unlike in the case of cationic gold complexes, these reaction conditions are likely to be more sensitive to steric factors in the case of AuNPs, since there are multiple gold species adjacent to the cationic Au site activated by oxygen. Therefore, the reaction conditions were expected to allow chemoselective hydroamination of terminal alkenes. The use of benzyl-protected amine (**1e**) resulted in the decomposition of the starting material. This is probably due to the unstable nature of benzylamine to the oxidising ability of the AuNPs/O_2_ condition.

**Figure 4:**
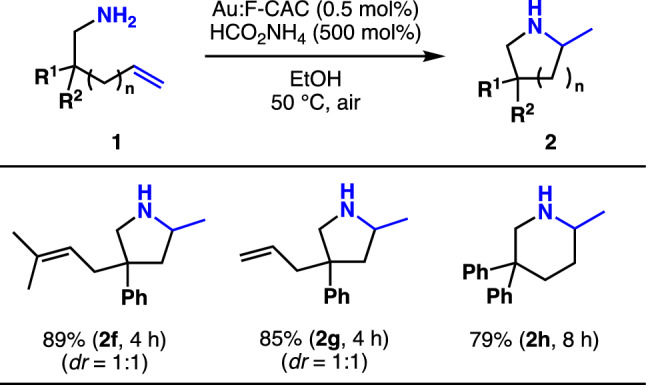
The substrate scope of intramolecular cyclisation of primary amine. *dr* diastereomeric ratio.

The substrates having different substituents at the β-position of the amino group were investigated (Fig. 4). As expected from the result that the reaction conditions are sensitive to the steric hindrance of alkene moiety, the chemoselective cyclisation of an amine with biased olefins is an excellent example of this unique system. The cyclisation of an amine having both allyl and prenyl group (**1f**) was subjected to this reaction to find that the cyclisation occurred at the allyl site to give **2f** (89%) with complete chemoselectivity (dr = 1:1). The reaction of an amine having a diallyl group (**1g**) also provides desired product **2g** (85%) within 4 h. The reaction proceeded smoothly when the amine-bearing homoallyl group (**1h**) was used, affording piperazine 2 h in 79%.

**Figure 5:**
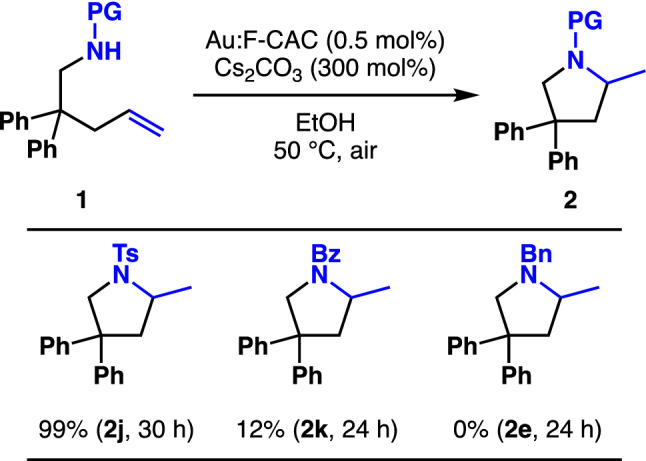
The substrate scope of protected amines.

We then applied the reaction conditions to the amines having protecting groups, such as toluenesulfonyl (**1j**) and benzoyl (**1k**) groups; however, it was found that the cyclisation reaction did not occur. After several attempts, we found that caesium carbonate (Cs_2_CO_3_) was adequate for the intermolecular cyclisation reaction to increase the nucleophilicity of amides. Pyrrolidine **2j** was obtained in quantitative yield when the sulfonimines **1j** were treated with 0.5 mol% of Au:F-CAC and 300 mol% of Cs_2_CO_3_ under an aerobic atmosphere at 50 °C for 30 h (Fig. 5). Although amide (**1k**) participated in this reaction, albeit in low yield (12%), the reaction did not proceed in the case of benzylamine **1e**, even under the reaction conditions.

## Conclusions

In summary, the Au:F-CAC catalyst shows high catalytic activity for the hydroamination reaction. The concentration of Au can be reduced up to 0.2 mol%. By focusing on the fact that this catalyst is substantially affected by the substitution pattern of alkene moiety, we achieved unprecedented chemoselective cyclisation, which has been difficult to perform in conventional cationic Au-catalysed reactions, affording novel compounds. Further investigations, such as application for the synthesis of fine chemicals and reaction developments, are in due course.

## Supplementary Information

Supplementary Information.

## Data Availability

The main data are available in the main text or the Supplementary Information. All other data are available from the authors upon reasonable request.
