# Supplementary material for: Intramolecular hydroamination catalysed by gold nanoparticles deposited on fibrillated cellulose
Source: Sci Rep. 2022 Nov 29;12:20602. doi: 10.1038/s41598-022-24955-3 (PMC9708665; doi:10.1038/s41598-022-24955-3)

Supporting Information

Intramolecular hydroamination catalysed by gold nanoparticles deposited on fibrillated cellulose

Yuta Uetake,[†,‡] Butsaratip Suwattananuruk,[†] Hidehiro Sakurai[†,‡,*]

[†]*Division of Applied Chemistry, Graduate School of Engineering, Osaka University*
2-1 Yamadaoka, Suita, Osaka 565-0871, Japan

[‡]*Innovative Catalysis Science Division, Institute for Open and Transdisciplinary Research Initiatives (ICS-OTRI), Osaka University, 2-1 Yamadaoka, Suita, Osaka 565-0871, Japan*

hsakurai@chem.eng.osaka-u.ac.jp

Contents

Instrumentations and chemicals S3–4

Preparation of Au:F-CAC catalysts S5–7

Optimization of reaction conditions S8–10

Synthetic procedure and compound data S11–16

References S17

NMR spectra S18–26

Instrumentations and Chemicals

All manipulations of moisture or air sensitive compounds were performed by standard Schlenk techniques in anhydrous solvents under nitrogen atmosphere using flame-dried glasswares. Reactions were conducted in an EYELA PPS-2511 personal organic synthesizer. Analytical thin-layer chromatography (TLC) was performed on pre-coated silica-gel aluminum sheets (Merck silica gel 60 F254, Cat. No. 1.05554.0001). Column chromatography was conducted on a YAMAZEN automated flash chromatography system that consists of an AI-580S and a Parallel Frac FR-360 using silica-gel (Kanto Chemical Co., Inc. Silica Gel 60 N (spherical, neutral)). Preparative thin-layer chromatography (PTLC) was prepared using Wako Wakogel B-5F. Organo Puric-ω water purification system was used to produce Ultrapure water (18.2 $\Omega\cdot cm^{-1}$). KUBOTA 7780II high speed refrigerated centrifuge attached with a RS-2504GS swing rotor was used to perform centrifugal ultrafiltration. EYELA FDU-2200 freeze drying system with a 10-station manifold was used for the freeze drying.

^{1}H NMR (400 MHz) and ^{13}C NMR (100 MHz) spectra were measured on a JEOL JNM-ECZS400 spectrometer at room temperature. Chloroform-d_1 ($CDCl_3$) was used as a solvent for NMR measurements. Chemical shifts (δ) are given in parts per million (ppm) downfield from the solvent signal (for ^{1}H NMR: $CHCl_3$ δ 7.26 ppm; for ^{13}C NMR: $CDCl_3$ δ 77.0 ppm) as an internal standard with coupling constants (*J*) in hertz (Hz).

High resolution mass spectra (HRMS) were measured on a JEOL JMS-700 using fast atom bombardment (FAB) ionization mode.

Infrared (IR) absorption spectra were measured by attenuated total reflection (ATR) method on a JASCO FT/IR-4100 Fourier transform IR spectrometer equipped with a JASCO ATR PRO ONE single reflection ATR optical attachment and a diamond crystal plate. The absorption bands were given in wavenumber (cm^{-1}).

JEOL JEM-2100 electron microscope at an accelerating voltage of 200 kV with using a Holey carbon support films coated Cu microgrid (EMJapan, U1003) was used to recorded Transmission electron microscopy (TEM) images. The TEM grid was applied hydrophilic treatment in a glow discharge irradiation chamber before use. Image-J software was used to analyze the generated TEM images, and the expressed mean diameter and standard deviation are based on an average of 300 particles.

Induced coupling plasma-atomic emission spectroscopy (ICP-AES) was performed on a Shimadzu ICPS-7510 emission spectrometer.

Unless otherwise noted, all reagents purchased from commercial suppliers were used without further purification.

Tetrachloroauric acid ($HAuCl_4 \cdot 4H_2O$) was purchased from Tanaka kikinzoku Kogyo K. K. Aqueous $HAuCl_4 \cdot 4H_2O$ solution (25 mmol/L) was prepared by dilution with aqueous HCl solution (40 mmol/L). Exact concentration of aqueous $HAuCl_4$ solution was determined by ICP-AES measurement prior to use. PVP(K-30) (Mw: 40,000 kDa), sodium borohydride ($NaBH_4$), hydrochloric acid (ca. 12 mol/L), ethanol (99.5%), acetic acid (CH_3CO_2H), aqueous ammonia solution (NH_3, 28%) and cellulose powder (Lot No. MOF2321) were purchased from Nacalai Tesque, Inc.

1,1,2,2-Tetrachloroethane, 3-chloro-2-metyl-1-propene, and 1-chloro-3-metyl-2-butene were purchased from Tokyo Chemical Industry Co., Ltd.

Buffer solution standard (phthalate pH standard solution) pH 4.01 and Ammonium formate were purchased from Kishida Chemical Co., Ltd.

Tetrahydrofuran (Dehydrated), Lithium aluminum hydride (>92.0%) was purchased from Kanto Chemical Co., Inc.

Cesium carbonate and diphenylacetonitrile were purchased from Sigma–Aldrich Japan Inc.

Chloroform-d_1 ($CDCl_3$) containing 0.05% tetramethylsilane (TMS) (99.8%D) was purchased from Cambridge Isotope Laboratories, Inc.

Citric acid was purchased FUJIFILM Wako Pure Chemical Corporation.

2,2-Diphenylpent-4-en-1-amine (**1a**),[1] 1-amino-2,2-diphenyl-4-methylpent-4-ene (**1b**),[2] 2,2-diphenylhex-4-en-1-amine (**1c**),[1] 1-amino-2,2-diphenyl-5-methylhex-4-ene (**1d**),[2] *N*-benzyl-2,2-diphenylpent-4-en-1-amine (**1e**),[3] 2-allyl-2-phenylpent-4-en-1-amine (**1g**),[4] 2,2-diphenylhex-5-en-1-amine (**1h**),[5] *N*-(2,2-diphenylpent-4-enyl)-4-methylbenzenesulfonamide (**1j**),[6] *N*-(2,2-diphenylpent-4-enyl)benzamide (**1k**)[1] were prepared according to the literatures.

Preparation of Au:F-CAC catalysts

Preparation of F-CAC (ca. 0.9 wt% citric acid loading)

Fibrillated citric acid-modified cellulose (F-CAC) was prepared according to the procedure with minor modifications.[7]

To a 500 mL round-bottomed flask were added cellulose (30 g) and distilled water (300 mL). To the suspension was added citric acid (90 g), and then the mixture was stirred for 5 min at room temperature. The mixture was transferred to a heat-resistant dish and incubated in an oven at 130 °C for 13 h. After cooling to room temperature, the residual citric acid was removed by washing with water until the pH of filtrate reached approximately 7. After that, the modified cellulose was successively washed with methanol (300 mL), acetone (300 mL), and then dried under vacuum to give CAC (ca. 0.9 wt% citric acid loading). Then, the thus-prepared CAC powder (20 g) was dispersed in water (200 mL) and to this was added an aqueous solution of NaOH (1 mol/L) to adjust the pH to 9. The solid was washed with water until the pH of filtrate reached approximately 7. Then, the solid was dispersed in water (400 mL) and fibrillated using a mixer (Vitamix, TNC5200, EntreX Incorporated, Tokyo, Japan) (15 sec × 50). All sample was transferred to a 1000 mL of round bottom flask and freeze dried for 2 days. After that, the solid was crushed by a mixer (15 sec × 50) to give F-CAC as a pale-yellow powder.

Figure S1. Schematic of the preparation procedure of F-CAC

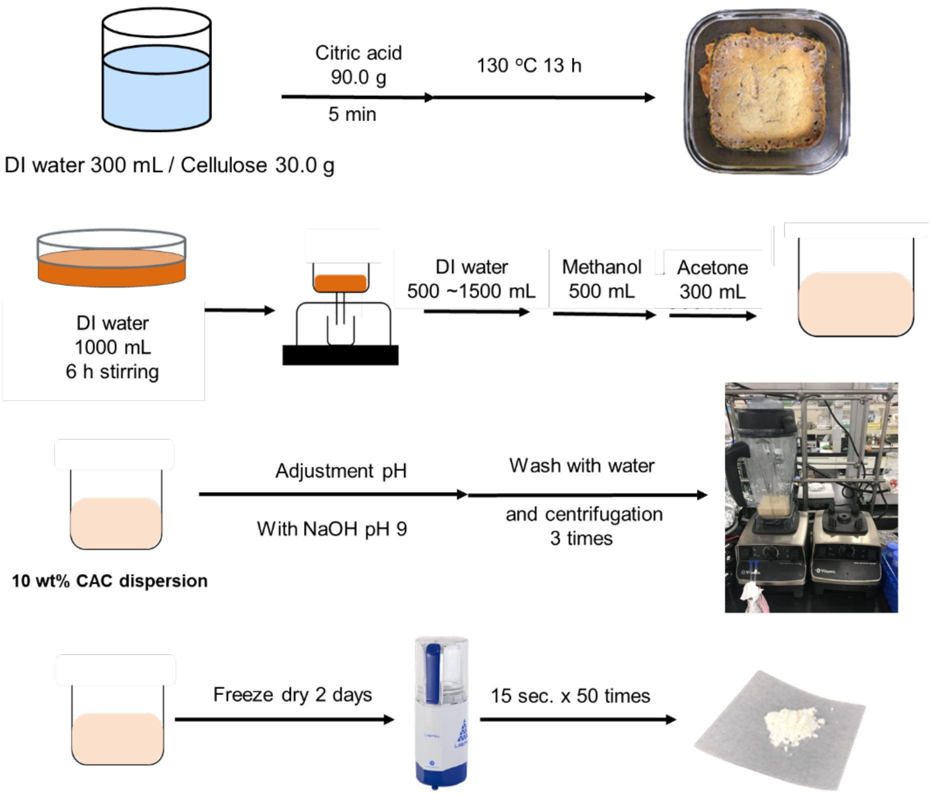

Titration of the amount of acids loaded[7]

Citric acid loading (mmol/g) was determined by conductimetric titration. F-CAC (500 mg) was suspended in water (150 mL) and added with 0.1 mol/L hydrochloric acid solutions until the pH reached 3. After 10 min of stirring, the suspension was titrated with 0.05 mol/L aqueous solution of NaOH with stirring. The conductivity was monitored using a conductivity meter throughout the titration process. The titration was terminated when the pH reached 11. Citric acid loading (mmol/g) was calculated by the following equation

$$\text{Citric acid loading (mmol/g)} = \frac{\text{(Concentration of NaOH)} \times \text{(Volume of NaOH)}}{\text{Cellulose (g)}}$$

Preparation of Au:F-CAC

Au:F-CAC was prepared according to the literature with modification.[8]

In a reaction tube (φ = 3 cm) equipped with a magnetic stir bar, Au:PVP(K-15) (5.1×10^{-3} mmol of Au) and F-CAC (600 mg) were mixed in ETOH(30 mL), and then the pH was adjusted to 4 using aqueous hydrochloric acid solution (0.1 mol/L). After stirring at 27 °C (1300 rpm) for 90 min, the solid was separated from the supernatant by centrifugation (7500 rpm) at room temperature and washed with ethanol (ca. 30 mL × 3). The remaining powder was dried under vacuum at 45 °C for 12 h to afford Au:F-CAC (1.67×10^{-3} wt%).

Figure S2. TEM image of Au:F-CAC

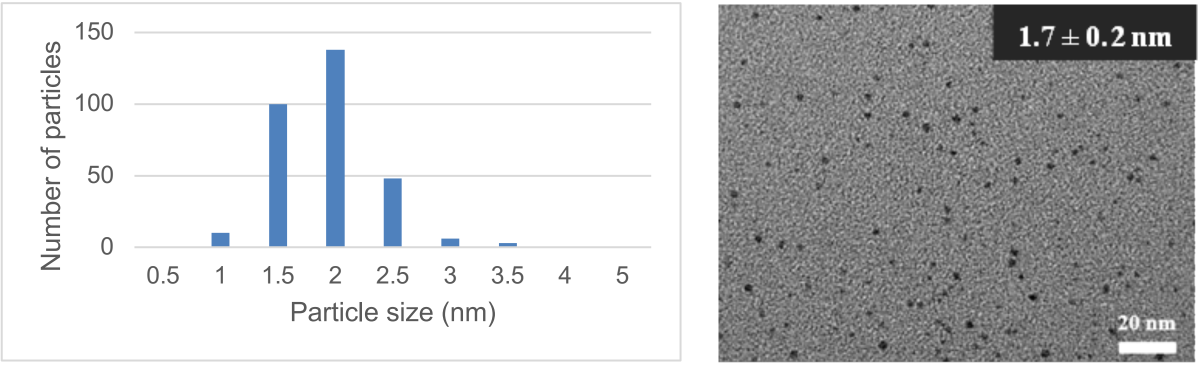

Sample preparation for ICP-AES measurement

ICP-AES measurement of amount of gold contain in Au:F-CAC

To a 10 mL volumetric flask containing an appropriate amount of Au:F-CAC was added a fleshly prepared aquaresia, and this was gently agitated by hand. After standing for 10 min, to this was added 0.1 mol/L aqueous hydrogen chloride solution. After fitting a ground glass stopper, the solution was mixed with inversion and passed through a membrane filter. ICP measurements were performed using an aliquot of this solution to determine an amount of gold contain in Au:F-CAC.

ICP-AES measurement of amount of gold leaching after reaction

To a 10 mL volumetric flask, 500 μL of filtrate (after reaction the mixture was filter to get rid of Au:F-CAC to obtain filtrate) was added. To this was added 0.1 mol/L aqueous hydrogen chloride solution. After fitting a ground glass stopper, the solution was mixed with inversion, and then ICP measurements were performed using an aliquot of this solution to determine an amount of gold leached after reaction (see Table 1 in main manuscript).

**Entry**	**Solvent**	**Additive**	**Leaching of gold (%)**
4	buffer/EtOH	HCO_2H (1000 mol%)<br>aq. NH_3 (2000 mol%)	45
5	EtOH	HCO_2H (1000 mol%)<br>aq. NH_3 (2000 mol%)	25

Optimization of reaction conditions

General procedure for hydroamination reaction

To a reaction tube equipped with a magnetic stir bar were added Au:F-CAC, amine (**1**) (0.10 mmol), additive, and solvent. The mixture was stirred under an ambient atmosphere. After stirring for a specific period, the catalyst was removed by filtration and washed with diethyl ether (ca. 5 mL × 3). the filtrate was evaporated to remove all solvent. Then to the drying sample was added 1,1,2,2-tetrachloroethane (10.2 μL, 0.10 mmol) as an internal standard. The mixture was diluted with $CDCl_3$, and ^1H NMR measurements were performed to determine the yields.

Table S1: Optimization of the ratio between HCO_2H and NH_3

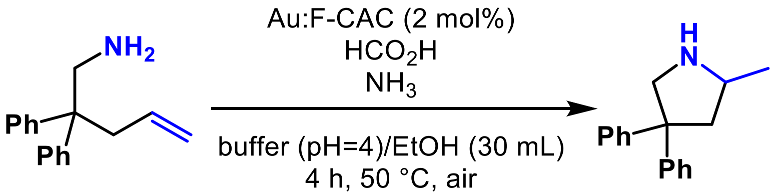

1a (0.1 mmol) **2a**

HCO_2H (mol%)	aq. NH_3 (mol%)	yield (%)[a]	
		**1a**	**2a**
1000	500	64	32
1000	1500	58	40
1000	2000	48	53
1000	4000	43	50
1000	6000	68	30
2000	4000	42	53
4000	8000	68	34

[a]Determined by ^1H NMR analysis.

Table S2: Optimization of the amount of HCO_2NH_4

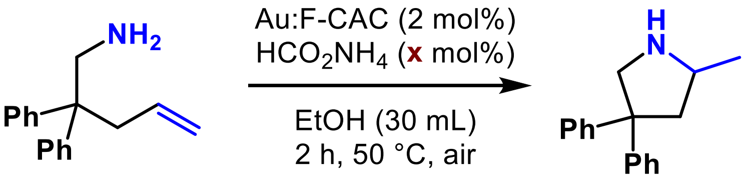

1a (0.1 mmol) **2a**

x	Yield (%)[a]	
	**1a**	**2a**
1000	7	90
500	8	85
400	20	79
200	55	44

[a]Determined by ^{1}H NMR analysis.

Table S3: Optimization of the amount of solvent

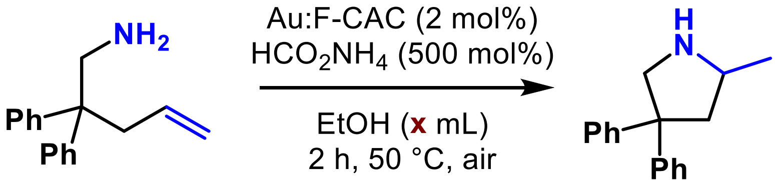

1a (0.1 mmol) **2a**

Amount of solvent (mL)	Yield (%)[a]	
	**1a**	**2a**
10	11	86
20	11	84
30	8	85

[a]Determined by ^{1}H NMR analysis.

Table S4: Optimization of Temperature

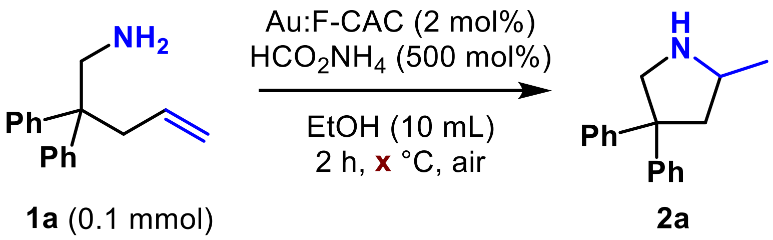

Temperature (°C)	Yield (%)[a]	
	**1a**	**2a**
60	0	100
50	11	86
40	58	41
27	77	22

[a]Determined by ^{1}H NMR analysis.

Table S5: Optimization of amount of gold

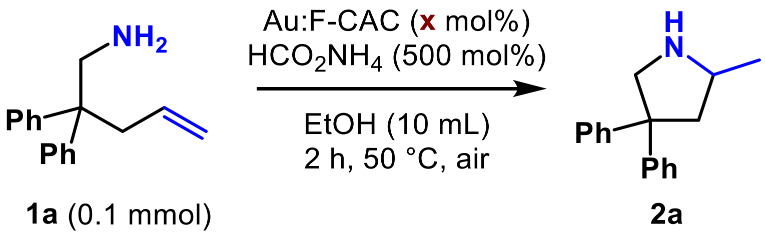

Amount of gold (mol%)	Yield (%)[a]	
	**1a**	**2a**
2	0	100
1	0	100
0.5	8	85
0.2	58	41

[a]Determined by ^{1}H NMR analysis.

Synthetic procedure and compound data

2-Phenylpent-4-enenitrile (1f-1)

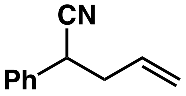

1f-1 was prepared according to the literature procedure.[1]

To a solution of *N*,*N*-diisopropylamine (3.1 mL, 22 mmol) in THF (12.5 mL) was added *n*-BuLi (8.0 mL, 21 mmol, 2.6 mol/L in *n*-hexane) at –78 °C. After stirring for 2 h, to this was added benzyl cyanide (2.1 mL, 18 mmol, 1 equiv) and the mixture was stirred for 3 h at the same temperature. To this was added 3-bromo-1-propene (1.7 mL, 20 mmol), and the reaction mixture was warmed to room temperature and stirred for 12 h at the same temperature. To this was added saturated aqueous NH_4Cl solution (50 mL) and the resulting mixture extracted with dichloromethane (50 mL × 3). The combined organic extract was dried over Na_2SO_4. After filtration, the filtrate was concentrated under reduced pressure. The residue was purified by column chromatography (*n*-hexane/EtOAc = 20:1) to give **1f-1** (2.6 g, 16.5 mmol, 82%);

R_f = 0.45 (*n*-hexane/EtOAc = 9:1);

^{1}H NMR ($CDCl_3$) δ 7.41−7.31 (m, 5H), 5.84–5.75 (m, 1H), 5.21–5.20 (m, 1H), 5.17–5.16 (m, 1H), 3.87 (dd, *J* = 7.8, 6.4 Hz, 1H), 2.66–2.62 (m, 2H);

^{13}C NMR ($CDCl_3$) δ 135.1 (1C), 132.5 (1C), 128.9 (2C), 128.0 (1C), 127.2 (2C), 120.2 (1C), 119.2 (1C), 39.7 (1C), 37.3 (1C);

The chemical shifts were consistent with those reported in the literature.[1]

2-Allyl-5-methyl-2-phenylhex-4-enenitrile (1f-2)

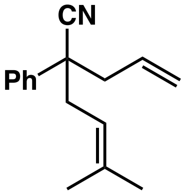

To a solution of *N*,*N*-diisopropylamine (2.9 mL, 21 mmol) in THF (50 mL) was added *n*-BuLi (8.0 mL, 21 mmol, 2.6 mol/L in *n*-hexane) at –78 °C. After stirring for 2 h, to this was added **1f-1** (2.75 g, 17.5 mmol, 1 equiv) and the mixture was stirred for 3 h at the same temperature. To this was added 1-chloro-3-methyl-but-2-ene (2.4 mL, 21 mmol, 1.2 equiv), and the reaction mixture was warmed to room temperature and stirred for 12 h at the same temperature. To this was added saturated aqueous NH_4Cl (50 mL) and the resulting mixture extracted with dichloromethane (50 mL × 3). The combined organic extract was dried over Na_2SO_4. After filtration, the filtrate was concentrated under reduced

pressure. The residue was purified by silica-gel column chromatography (*n*-hexane/EtOAc = 4:1) to give **1f-2** (3.09 g, 13.7 mmol, 78%);
TLC R_f = 0.67 (*n*-hexane/EtOAc = 4:1);
^{1}H NMR ($CDCl_3$) δ 7.42–7.35 (m, 3H), 7.33–7.27 (m, 2H), 5.71–5.62 (m, 1H), 5.17–5.10 (m, 2H), 5.09–5.03 (m, 1H), 2.75–2.64 (m, 3H), 2.59–2.54 (dd, *J* = 14.6, 6.9 Hz, 1H), 1.67 (s, 3H), 1.55 (s, 3H);
^{13}C NMR ($CDCl_3$) δ: 138.1 (1C), 136.8 (1C), 131.9 (1C), 128.7 (2C), 127.6 (1C), 126.3 (2C), 122.1 (1C), 119.9 (1C), 117.4 (1C), 48.0 (1C), 43.8 (1C), 38.7 (1C), 25.8 (1C), 18.1 (1C);
IR (diamond, cm^{-1}) 3059, 2980, 2913, 2235, 1671, 1642, 1600, 1494, 1451, 1377, 1111;
HRMS (EI) *m/z* 226.1593 (226.1551 calcd for $C_{16}H_{19}N$, $[M+H]^+$).

2-Allyl-5-methyl-2-phenylhex-4-en-1-amine (1f)

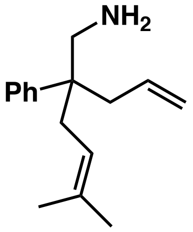

To a suspension of lithium aluminum hydride (0.65 g, 17 mmol) in Et_2O (30 mL) was slowly added a solution of **1f-2** (2.9 g, 13 mmol) in Et_2O (10 mL) at 0 °C under nitrogen atmosphere. The reaction mixture was allowed to warm to room temperature and stirred for 12 h at the same temperature. To the mixture were added Et_2O (30 mL), water (1.6 mL), 15% aqueous NaOH solution (1.6 mL), and water (5.0 mL). The combined organic extract was dried over Na_2SO_4. After filtration, the filtrate was concentrated under reduced pressure. The residue was purified by silica-gel column chromatography (CH_2Cl_2/MeOH/Et_3N = 49/49/2) to give **1f** (2.71 g, 11.8 mmol, 90%);
TLC R_f = 0.75 (CH_2Cl_2/MeOH/Et_3N = 49/49/2);
^{1}H NMR ($CDCl_3$) δ 7.38–7.29 (m, 4H), 7.24–7.18 (m, 1H), 5.67–5.56 (m, 1H), 5.10–4.93 (m, 3H), 2.91 (s, 2H), 2.53–2.31 (m, 4H), 1.65 (s, 3H), 1.58 (s, 3H);
^{13}C NMR ($CDCl_3$) δ: 144.6 (1C), 134.7 (1C), 133.3 (1C), 128.1(2C), 126.7(2C), 125.7 (1C), 119.6 (1C), 117.2 (1C), 48.9 (1C), 46.1 (1C), 39.8 (1C), 33.8 (1C), 25.9 (1C), 17.9 (1C);
IR (diamond, cm^{-1}); 3058, 2972, 2912, 2855, 1637, 1598, 1494, 1444, 1375, 1033;
HRMS (EI) *m/z* 229.1829 (229.1830 calcd for $C_{16}H_{23}N$, $[M+H]^+$).

2-Methyl-4,4-diphenylpyrrolidine (2a)

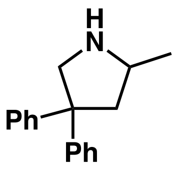

To a reaction tube equipped with a magnetic stir bar were added Au:F-CAC (0.5 mol%), amine (**1a**) (23.7 mg, 0.10 mmol), HCO_2NH_4 (31.5 mg, 500 mol%), and EtOH (10 mL). The mixture was stirred under air for 3 h at 50 °C. After cooling to room temperature, to the mixture was added saturated aqueous $NaHCO_3$ (ca. 5 mL), and the mixture was extracted with EtOAc. The combined organic extract was dried over Na_2SO_4. After filtration, the filtrate was concentrated under reduced pressure. The residue was purified by preparative TLC to give **2a** (23.5 mg, 0.99 mmol, 99%);
TLC R_f = 0.37 (CH_2Cl_2/MeOH/Et_3N = 49/49/2);
^{1}H NMR ($CDCl_3$) δ 7.31–7.15 (m, 10H), 3.68 (d, J = 11.4 Hz, 1H), 3.47 (d, J = 11.4 Hz, 1H), 3.38 (ddd, J = 8.9, 6.5, 6.4 Hz, 1H), 2.75 (dd, J = 12.7, 6.5 Hz, 1H), 2.04 (dd, J =12.7, 8.9 Hz, 1H), 1.66 (s, 1H), 1.21 (d, J = 6.4 Hz, 3H);
^{13}C NMR ($CDCl_3$) δ 147.7 (1C), 147.0 (1C), 128.31 (2C), 128.27 (2C) 127.0 (2C), 126.9 (2C), 126.0 (2C), 57.8 (1C), 57.3 (1C), 53.1, (1C), 47.0 (1C), 22.4 (1C);
The chemical shifts were consistent with those reported in the literature.[1]

2,2-Dimethyl-4,4-diphenylpyrrolidine (2b)

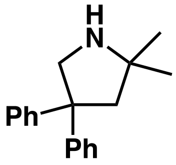

To a reaction tube equipped with a magnetic stir bar were added Au:F-CAC (1 mol%), amine (**2b**) (25.1 mg, 0.10 mmol), HCO_2NH_4 (31.5 mg, 500 mol%), and EtOH (10 mL). The mixture was stirred under air for 4 h at 50 °C. After cooling to room temperature, to the mixture was added saturated aqueous $NaHCO_3$ (ca. 5 mL), and the mixture was extracted with EtOAc. The combined organic extract was dried over Na_2SO_4. After filtration, the filtrate was concentrated under reduced pressure. The residue was purified by preparative TLC to give **2b** (24.8 mg, 0.99 mmol, 99%);
TLC R_f = 0.66 (CH_2Cl_2/MeOH/Et_3N = 70/38/2);
^{1}H NMR ($CDCl_3$) δ 7.32–7.24 (m, 8H), 7.18–7.12 (m, 2H), 3.66 (s, 2H), 2.55 (s, 2H), 1.84 (1s), 1.16 (s, 6H);
^{13}C NMR ($CDCl_3$) δ 147.5 (2C), 128.4 (4C), 126.9 (4C), 125.9 (2C), 59.3 (1C), 58.3 (1C), 57.2 (1C), 52.0 (1C), 30.7 (2C);

The chemical shifts were consistent with those reported in the literature.[2]

2-Methyl-4-(3-methylbut-2-en-1-yl)-4-phenylpyrrolidine (2f)

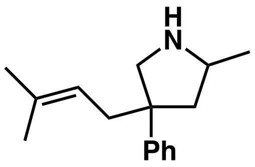

To a reaction tube equipped with a magnetic stir bar were added Au:F-CAC (0.5 mol%), amine (**2f**) (22.9 mg, 0.10 mmol), HCO_2NH_4 (31.5 mg, 500 mol%), and EtOH (10 mL). The mixture was stirred under air for 4 h at 50 °C. After cooling to room temperature, to the mixture was added saturated aqueous $NaHCO_3$ (ca. 5 mL), and the mixture was extracted with EtOAc. The combined organic extract was dried over Na_2SO_4. After filtration, the filtrate was concentrated under reduced pressure. The residue was purified by preparative TLC to give **2f** (20.4 mg, 0.89 mmol, 89%, dr = 1:1) as a diastereomeric mixture (dr = 1:1);

TLC R_f = 0.73 (CH_2Cl_2/MeOH/Et_3N = 90/8/2);

^{1}H NMR ($CDCl_3$) δ 7.32–7.15 (m, 5 H), 4.94–4.83 (m, 1H), 3.50 (ddd, J = 8.9, 6.5, 6.4 Hz, 0.5H), 3.30–3.12 (m, 2.5H), 2.44–2.24 (m, 3H), 1.61 (s, 3H), 1.58–1.49 (m, 1H), 1.44 (s, 1.5H), 1.41 (s, 1.5H), 1.24 (d, J = 6.4 Hz, 1.5H), 1.19 (d, J = 6.4 Hz, 1.5H);

^{13}C NMR ($CDCl_3$) δ 147.6 (0.5C), 147.3 (0.5C), 133.8 (0.5C), 133.6 (0.5C), 127.9 (0.5C+0.5C), 127.8 (0.5C+0.5C), 126.9 (1C+1C), 125.6 (0.5C+0.5C), 120.6 (0.5C), 120.4 (0.5C), 57.9 (0.5C), 57.1 (0.5C), 54.1 (0.5C), 53.3 (0.5C), 52.63 (0.5C), 52.57 (0.5C), 45.8 (0.5C), 45.2 (0.5C), 40.9 (0.5C), 39.3 (0.5C), 25.8 (0.5C+0.5C), 22.0 (0.5C), 21.9 (0.5C), 17.8 (0.5C), 17.7 (0.5C);

IR (diamond, cm^{-1}) 2960, 2922, 2854, 1605, 1496, 1442, 1401, 1222, 1111, 1074, 1029;

HRMS (EI) m/z 229.1822 (229.1830 calcd for $C_{16}H_{23}N$, $[M+H]^+$).

4-Allyl-2-methyl-4-phenylpyrrolidine (2g)

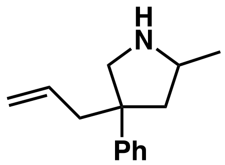

To a reaction tube equipped with a magnetic stir bar were added Au:F-CAC (0.5 mol%), amine (**2g**) (20.1 mg, 0.10 mmol), HCO_2NH_4 (31.5 mg, 500 mol%), and EtOH (10 mL). The mixture was stirred under air for 4 h at 50 °C. After cooling to room temperature, to the mixture was added saturated aqueous $NaHCO_3$ (ca. 5 mL), and the mixture was extracted with EtOAc. The combined organic extract was dried over Na_2SO_4. After filtration, the filtrate was concentrated under reduced pressure.

The residue was purified by preparative TLC to give **2g** (17.1 mg, 0.85 mmol, 85%) as a diastereomeric mixture (dr = 1:1);

TLC R_f = 0.62 (CH_2Cl_2/MeOH/Et_3N = 88/10/2);

^{1}H NMR (400 MHz, $CDCl_3$) δ 7.31–7.28 (m, 2H), 7.24–7.17 (m, 3H), 5.50–5.43 (m, 1H), 4.96–4.92 (m, 2H), 3.51–3.40 (m, 0.5H), 3.32–3.12 (m, 2.5H), 2.52–2.34 (m, 2.5H), 2.29 (dd, *J* = 12.4, 6.4 Hz, 0.5H), 1.61 (dd, *J* = 12.4, 9.2 Hz, 0.5H), 1.54 (dd, *J* = 13.3, 8.2 Hz, 0.5H), 1.23 (d, *J* = 6.4 Hz, 1.5H), 1.17 (d, *J* = 6.0 Hz, 1.5H);

^{13}C NMR ($CDCl_3$) δ 147.2 (0.5C), 147.0 (0.5C), 135.1 (0.5C), 134.9 (0.5C), 128.1 (1C), 128.0 (1C), 126.8 (2C), 125.9 (0.5C), 125.8 (0.5C), 117.4 (0.5C), 117.3 (0.5C), 57.9 (0.5C), 57.0 (0.5C), 54.1 (0.5C), 53.1 (0.5C), 52.0 (0.5C+0.5C, two signals overlapped), 47.3 (0.5C), 45.8 (0.5C), 45.6 (0.5C), 45.1 (0.5C), 22.3 (0.5C), 22.1 (0.5C);

The chemical shifts were consistent with those reported in the literature.[4]

2-Methyl-5,5-diphenylpiperidine (2h)

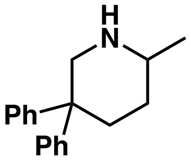

To a reaction tube equipped with a magnetic stir bar were added Au:F-CAC (0.5 mol%), amine (**2h**) (25.1 mg, 0.10 mmol), HCO_2NH_4 (31.5 mg, 500 mol%), and EtOH (10 mL). The mixture was stirred under air for 8 h at 50 °C. After cooling to room temperature, to the mixture was added saturated aqueous $NaHCO_3$ (ca. 5 mL), and the mixture was extracted with EtOAc. The combined organic extract was dried over Na_2SO_4. After filtration, the filtrate was concentrated under reduced pressure. The residue was purified by preparative TLC to give **2h** (19.8 mg, 0.79 mmol, 79%);

TLC R_f = 0.43 (CH_2Cl_2/MeOH/Et_3N = 88/10/2);

^{1}H NMR (400 MHz, $CDCl_3$) δ 7.41 (d, *J* = 7.32 Hz, 2H), 7.35 (t, *J* = 7.8 Hz, 2H), 7.24–7.01 (m, 6H), 3.92 (dd, *J* = 13.6, 3.0 Hz, 1H), 3.11 (d, *J* = 13.6 Hz, 1H), 2.83–2.73 (m, 1H), 2.71 (ddd, *J* = 13.5, 6.6, 3.4 Hz, 1H), 2.22 (ddd, *J* = 13.5, 13.5, 3.4 Hz, 1H), 1.64 (ddd, *J* = 13.5, 6.6, 3.4 Hz, 1H), 1.23–1.08 (m, 1H), 1.01 (d, *J* = 6.4 Hz, 1H);

^{13}C NMR ($CDCl_3$) δ 148.8 (1C), 144.7 (1C), 128.6 (2C), 128.2 (4C), 126.4 (2C), 125.78 (1C), 125.76 (1C), 55.7 (1C), 52.3 (1C), 45.2 (1C), 35.4 (1C), 31.4 (1C), 22.5 (1C);

The chemical shifts were consistent with those reported in the literature.[5]

2-Methyl-4,4-diphenyl-1-tosylpyrrolidine (2j)

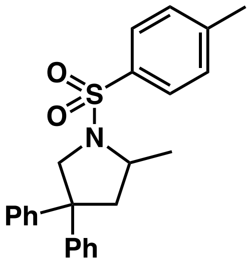

To a reaction tube equipped with a magnetic stir bar were added Au:F-CAC (0.5 mol%), amine (**2j**) (39.1 mg, 0.10 mmol), Cs_2CO_3 (97.7 mg, 300 mol%), and EtOH (10 mL). The mixture was stirred under air for 30 h at 50 °C. After cooling to room temperature, to the mixture was added saturated aqueous $NaHCO_3$ (ca. 5 mL), and the mixture was extracted with EtOAc. The combined organic extract was dried over Na_2SO_4. After filtration, the filtrate was concentrated under reduced pressure. The residue was purified by preparative TLC to give **2j** (38.6 mg, 0.99 mmol, 99%);

TLC R_f = 0.64 (CH_2Cl_2/MeOH/Et_3N = 67/30/3);

^{1}H NMR ($CDCl_3$) δ 7.62–7.60 (AA'BB', 2H), 7.33–7.01 (m, 12H), 4.17 (d, J = 10.5 Hz, 1H), 3.94 (d, J = 10.5 Hz, 1H), 3.78 (qdd, J = 7.3, 6.9, 6.4 Hz, 1H), 2.79 (dd, J = 12.3, 7.3 Hz, 1H), 2.39 (s, 3H), 2.26 (dd, J = 12.3, 6.9 Hz, 1H), 1.24 (d, J = 6.4 Hz, 3H);

^{13}C NMR ($CDCl_3$) δ 145.5 (1C), 144.8 (1C), 142.9 (1C), 135.3 (1C), 129.5 (2C), 128.4 (4C), 127.1 (2C), 126.7 (2C), 126.44 (2C), 126.41 (1C), 126.2 (1C), 58.4 (1C), 55.4 (1C), 52.2 (1C), 46.0 (1C), 22.1 (1C), 21.4 (1C);

The chemical shifts were consistent with those reported in the literature.[6]

References

1 A. Horn and P. H. Dussault, *J. Org. Chem.*, 2019, **84**, 14611–14626.

2 M. R. Crimmin, M. Arrowsmith, A. G. M. Barrett, I. J. Casely, M. S. Hill and P. A. Procopiou, *J. Am. Chem. Soc.*, 2009, **131**, 9670–9685.

3 L. Hussein, N. Purkait, M. Biyikal, E. Tausch, P. W. Roesky and S. Blechert, *Chem. Commun.*, 2014, **50**, 3862–3864.

4 L. Ferrand, Y. Tang, C. Aubert, L. Fensterbank, V. Mouriès-Mansuy, M. Petit and M. Amatore, *Org. Lett.*, 2017, **19**, 2062–2065.

5 H. Ohmiya, T. Moriya and M. Sawamura, *Org. Lett.*, 2009, **11**, 2145–2147.

6 G.-Q. Liu, W. Li and Y.-M. Li, *Adv. Synth. Catal.*, 2013, **355**, 395–402.

7 (a) X. Cui, T. Honda, T.-A. Asoh and H. Uyama, *Carbohydr. Polym.*, 2020, **230**, 115662.; X. Cui, A. Ozaki, T.-A. Asoh, H. Uyama, 2020, **175**, 109118.

8 T. Chutimasakul, Y. Uetake, J. Tantirungrotechai, T. Asoh, H. Uyama and H. Sakurai, *ACS Omega*, 2020, **5**, 33206–33213.

^{1}H NMR (400 MHz) and ^{13}C NMR (100 MHz) spectra of **1f-1**($CDCl_3$)

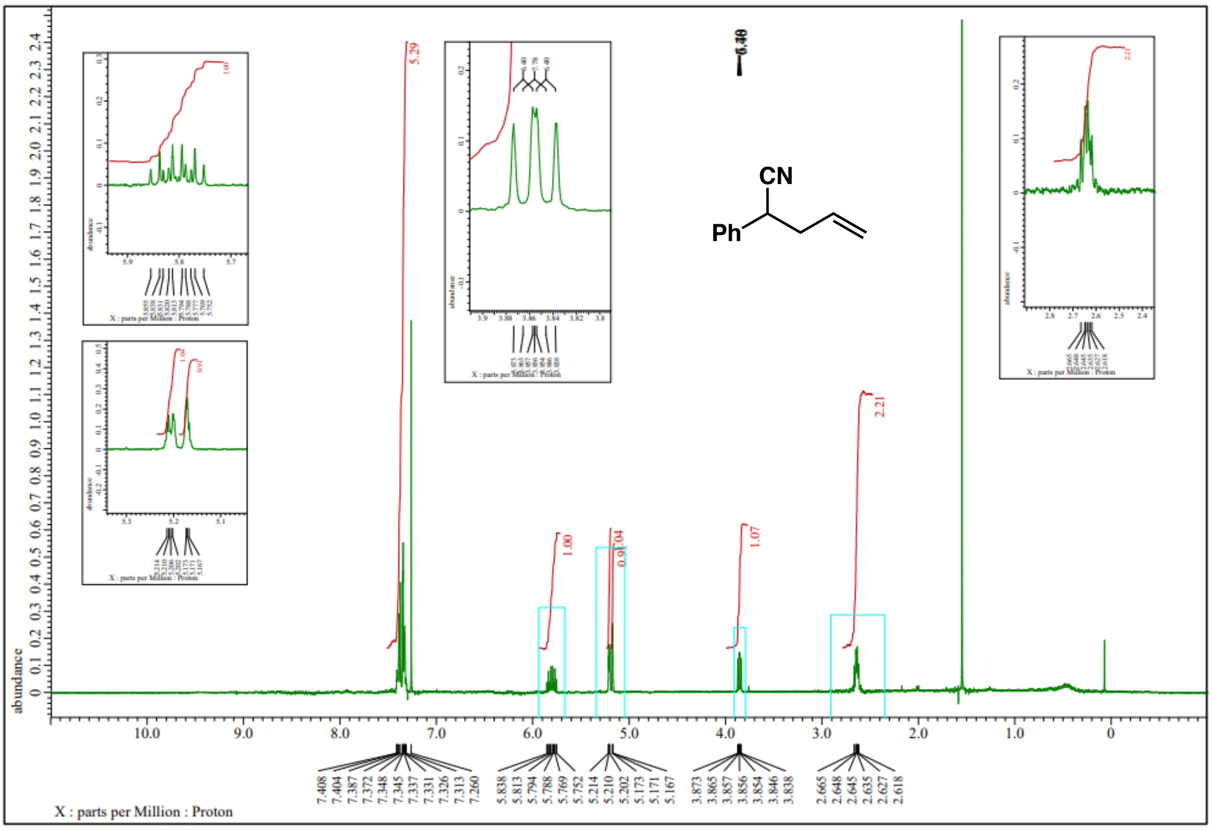

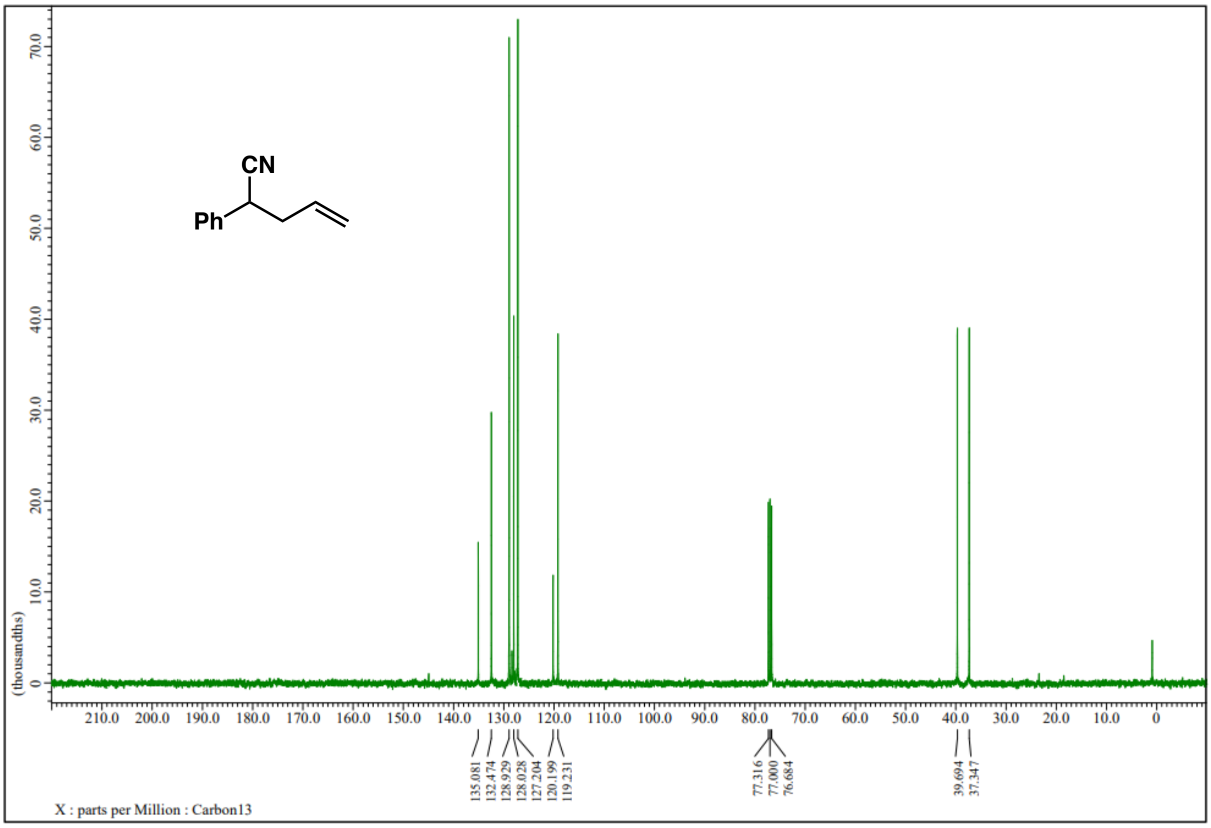

^{1}H NMR (400 MHz) and ^{13}C NMR (100 MHz) spectra of **1f-2**($CDCl_3$)

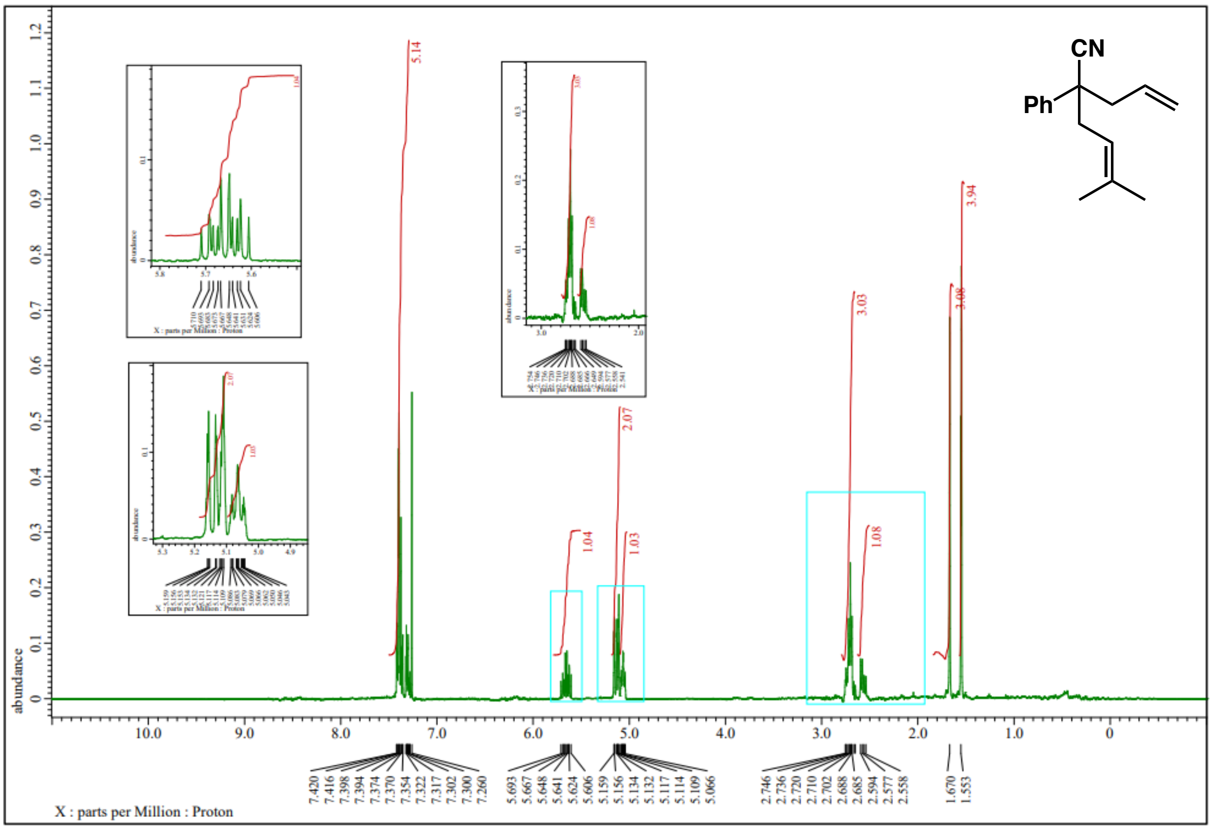

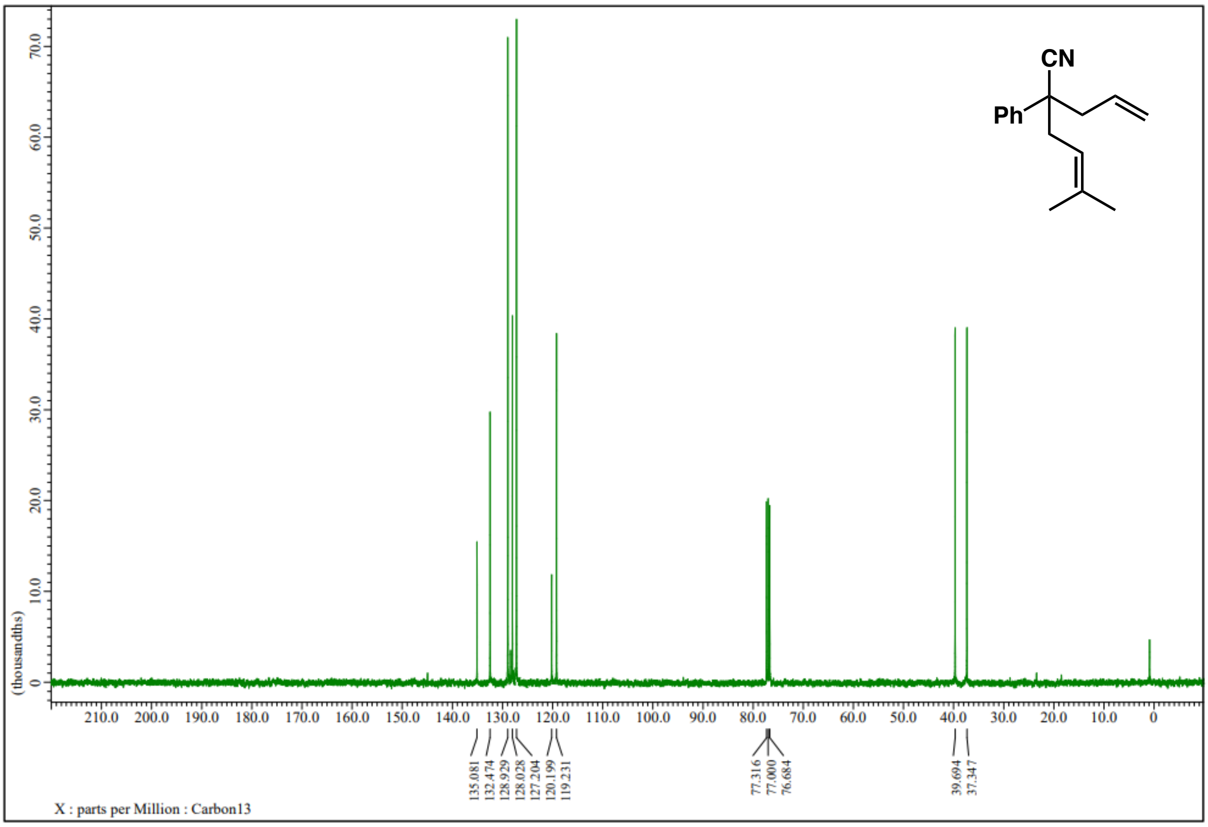

^{1}H NMR (400 MHz) and ^{13}C NMR (100 MHz) spectra of **1f** ($CDCl_3$)

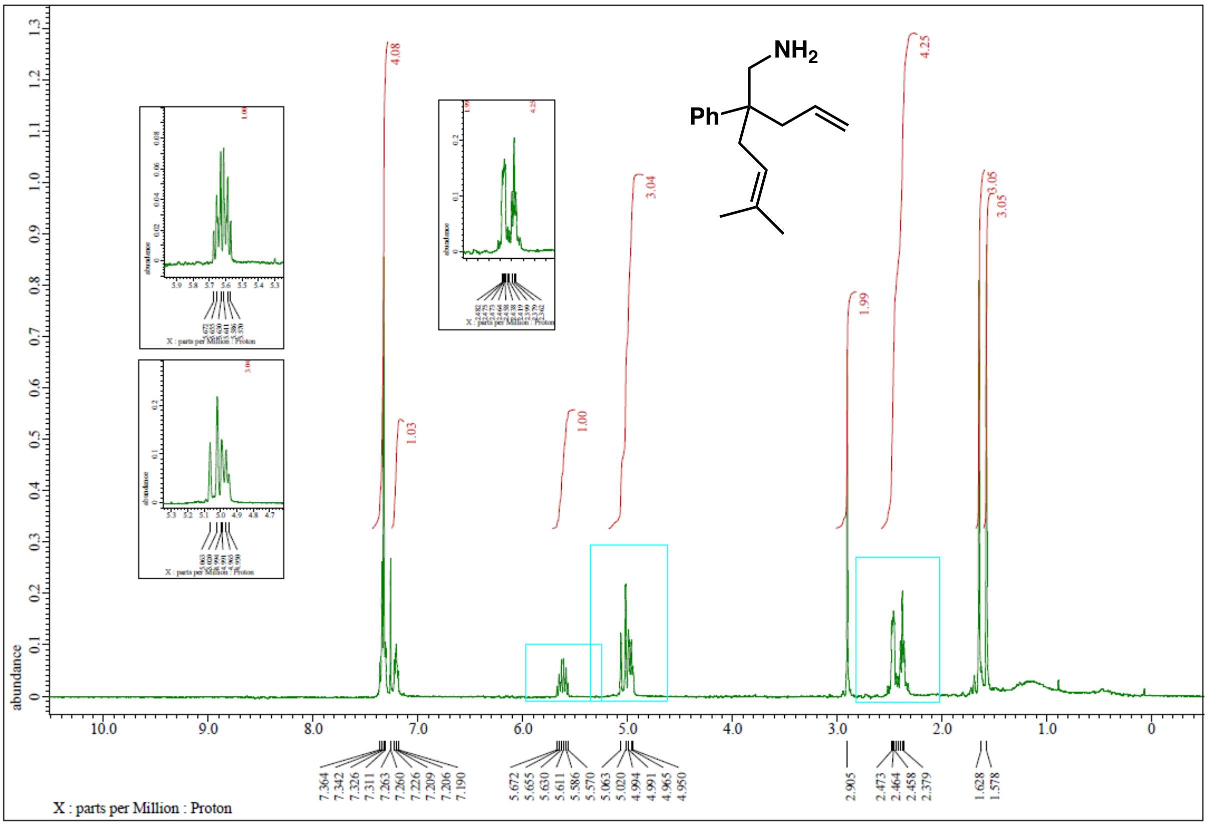

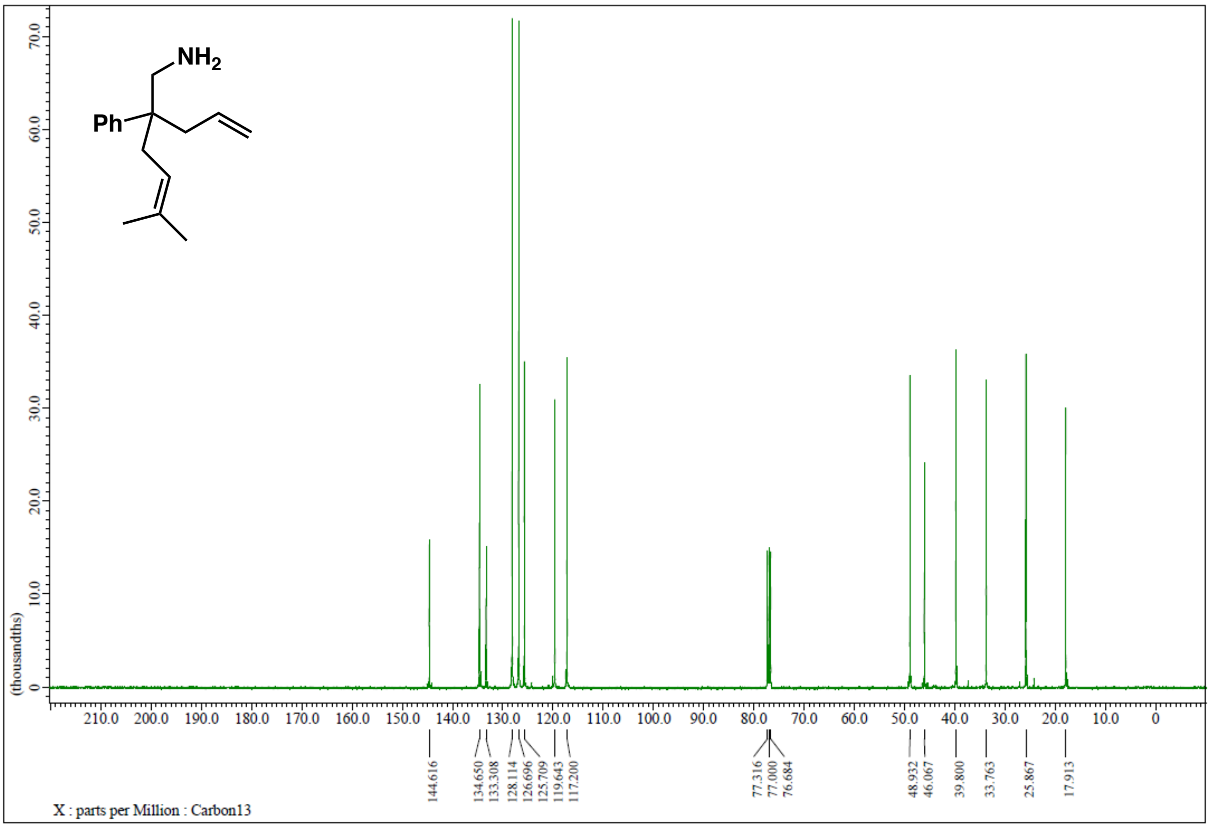

^{1}H NMR (400 MHz) and ^{13}C NMR (100 MHz) spectra of **2a** ($CDCl_3$)

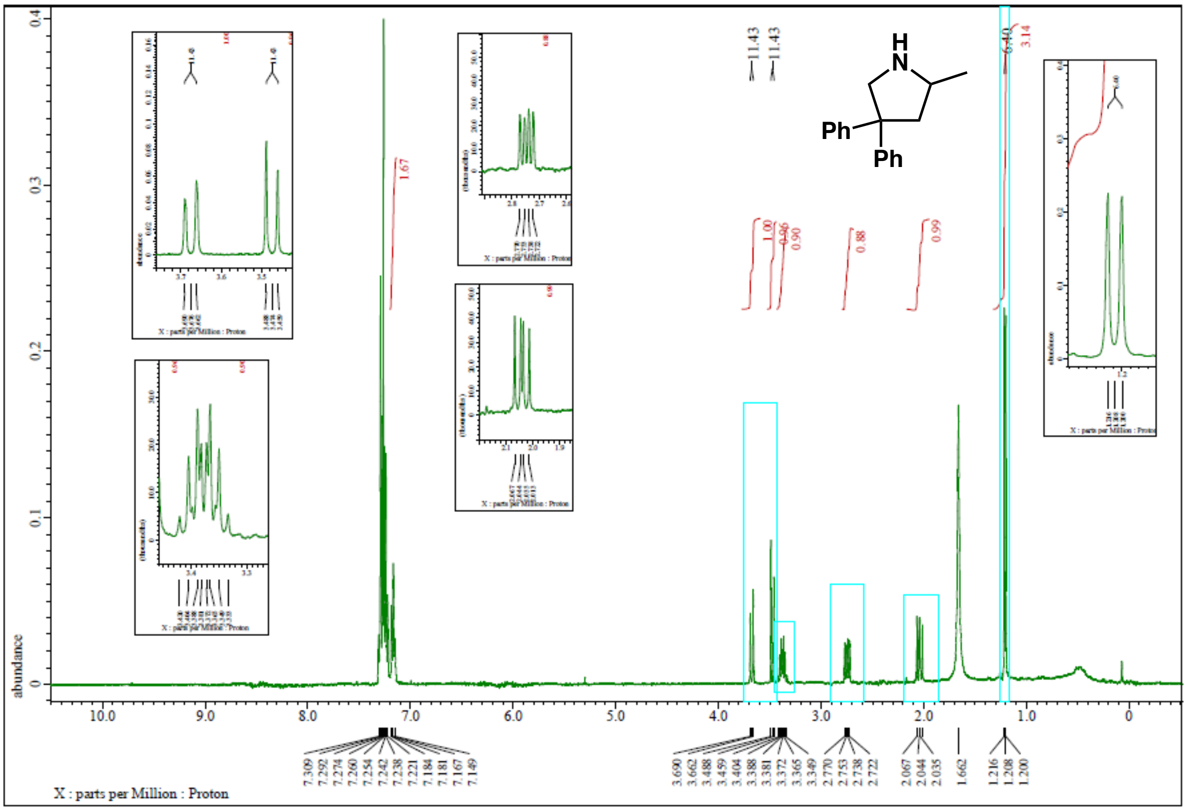

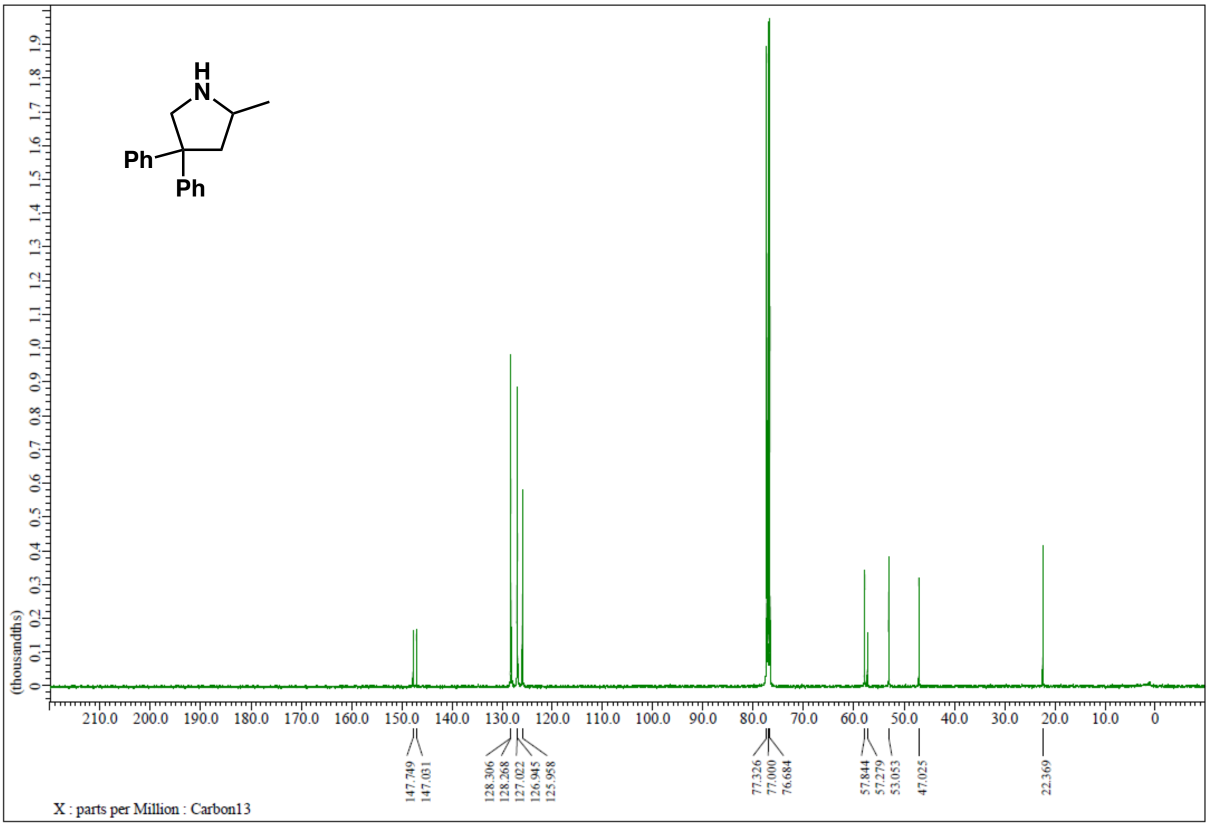

^{1}H NMR (400 MHz) and ^{13}C NMR (100 MHz) spectra of **2b**($CDCl_3$)

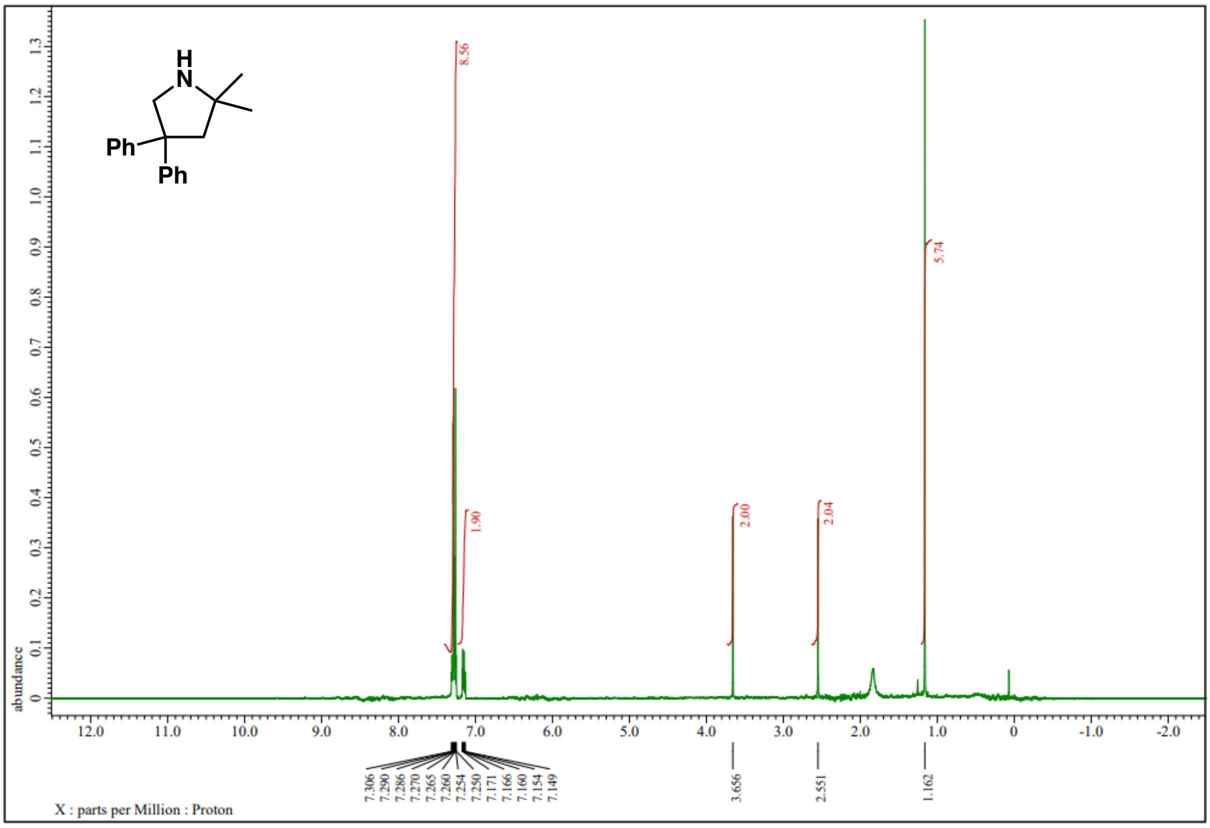

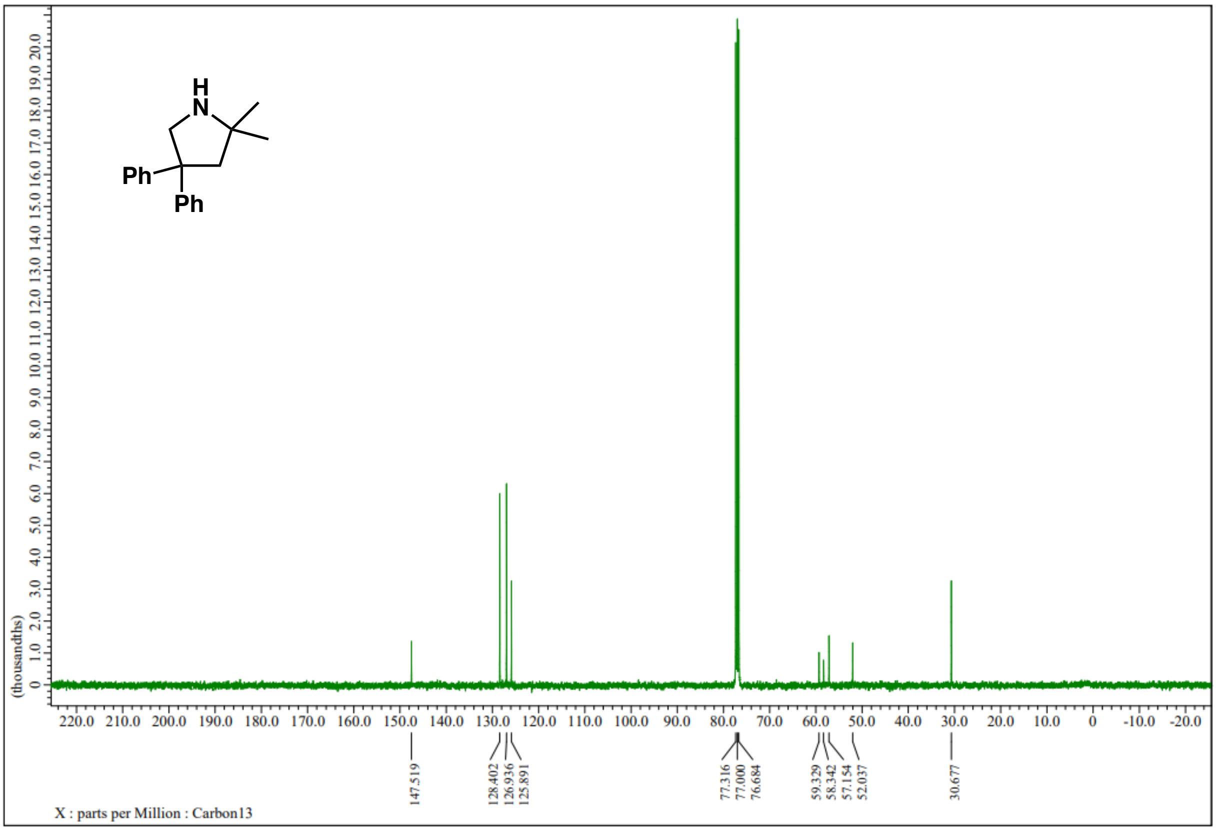

^{1}H NMR (400 MHz) and ^{13}C NMR (100 MHz) spectra of **2f** ($CDCl_3$)

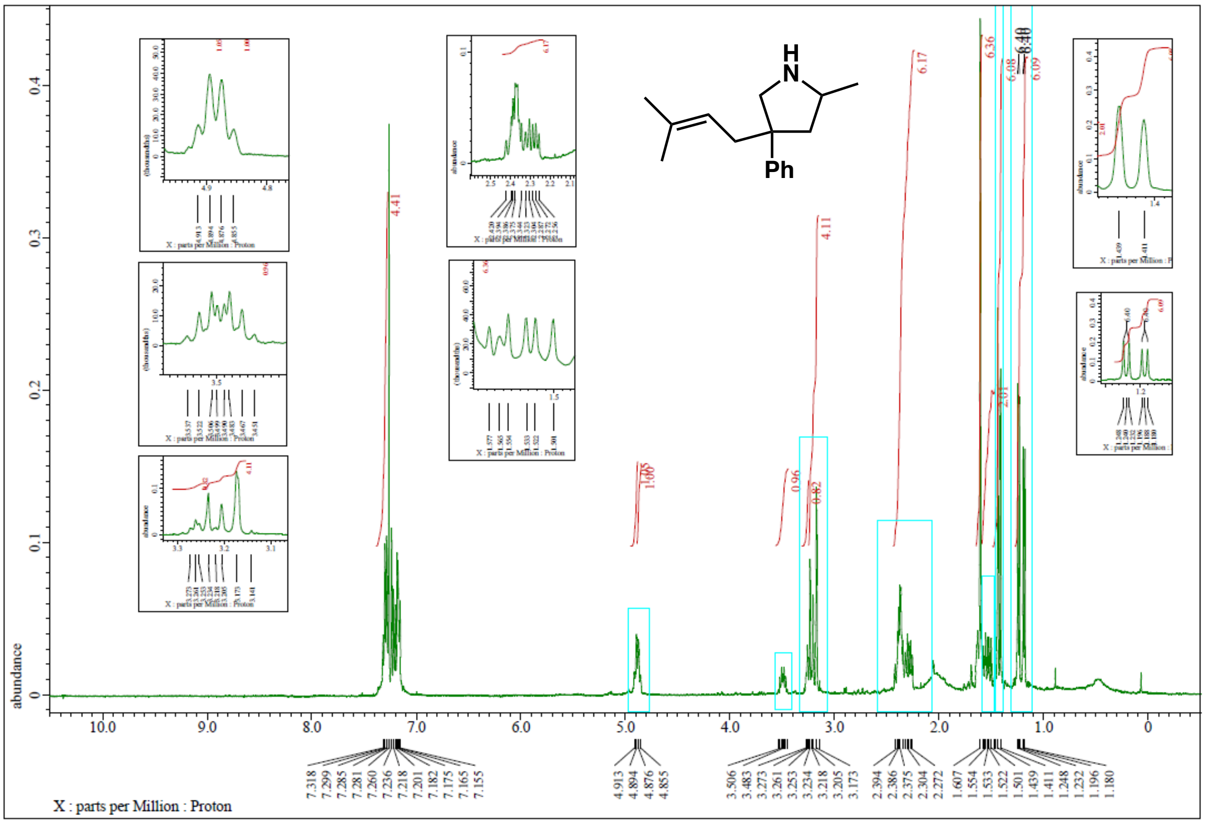

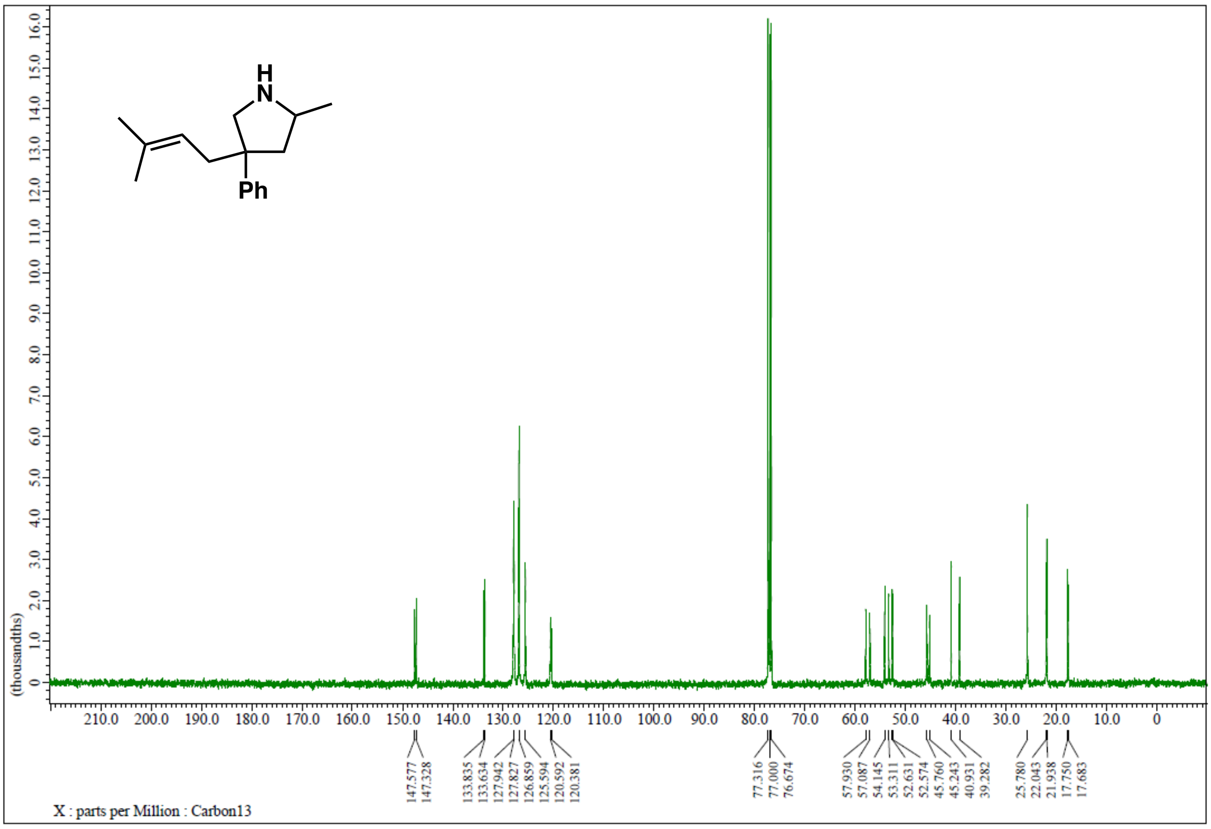

^{1}H NMR (400 MHz) and ^{13}C NMR (100 MHz) spectra of **2g** ($CDCl_3$)

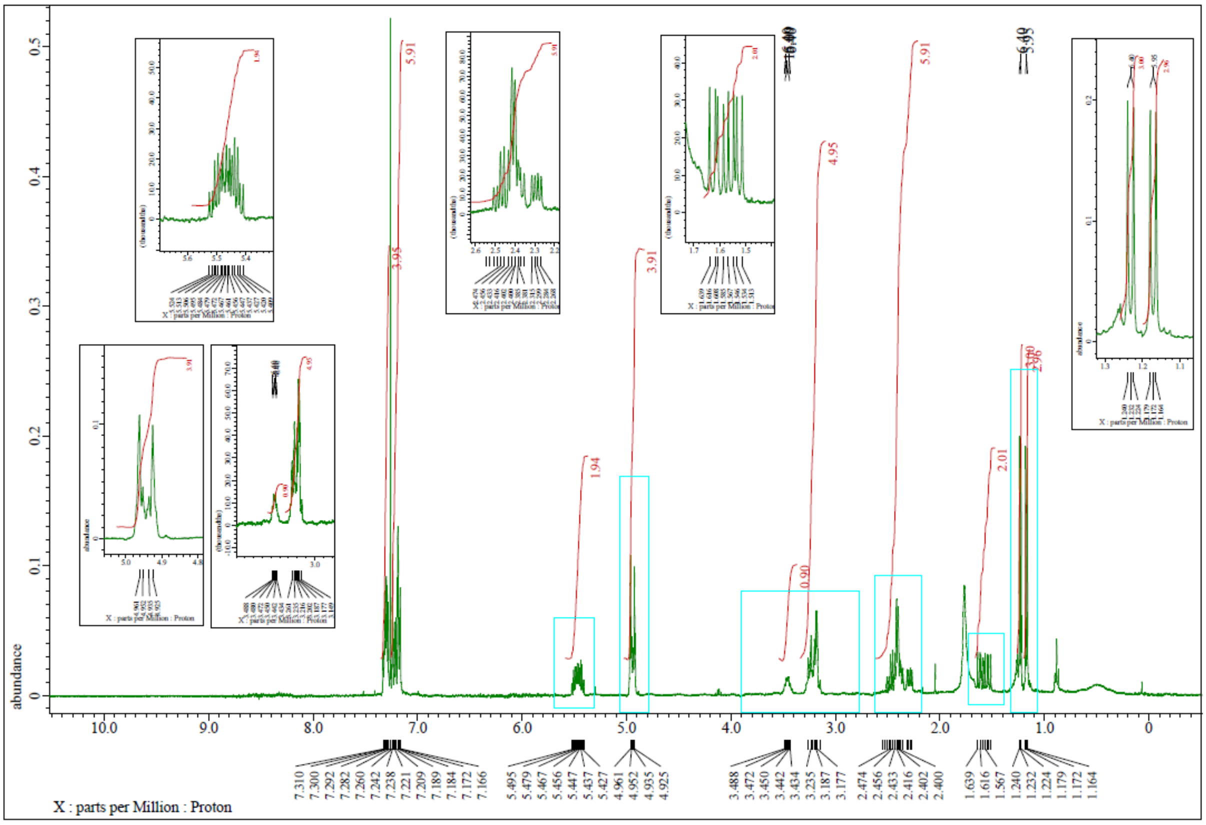

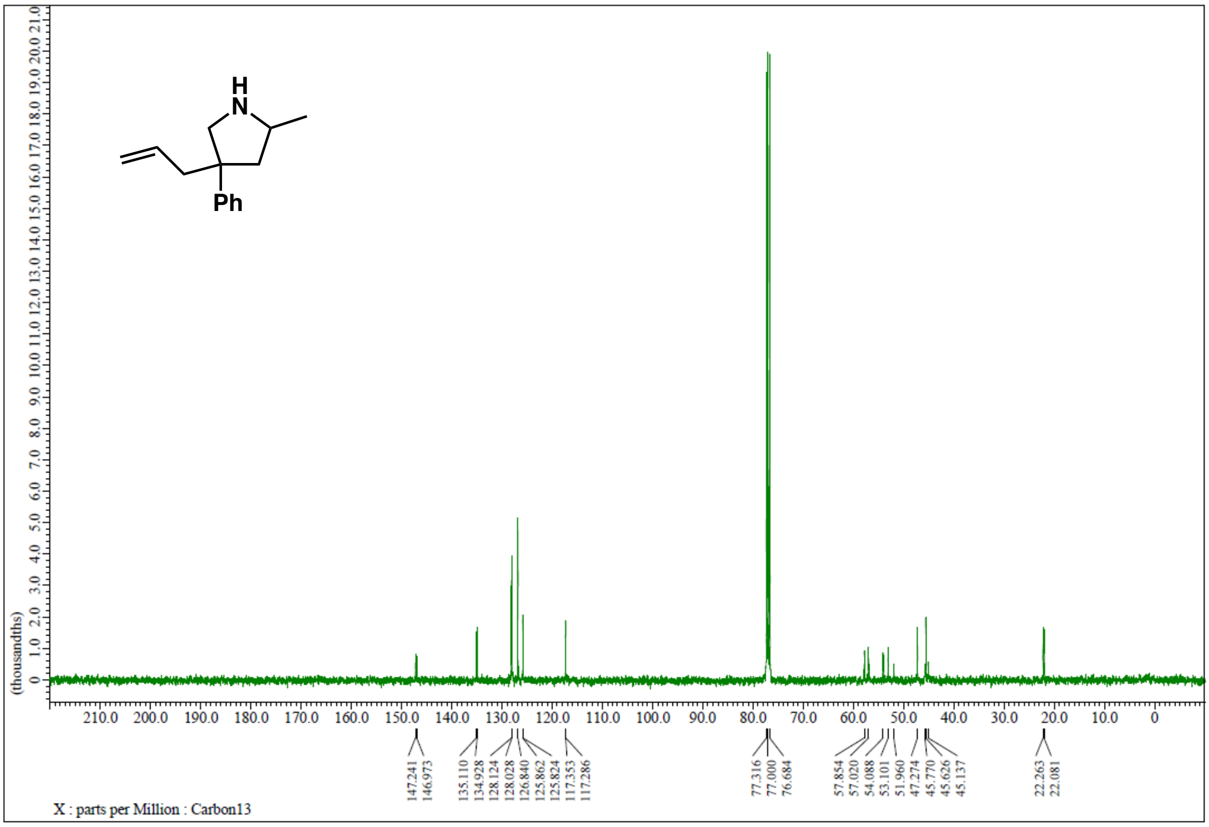

^{1}H NMR (400 MHz) and ^{13}C NMR (100 MHz) spectra of **2h** ($CDCl_3$)

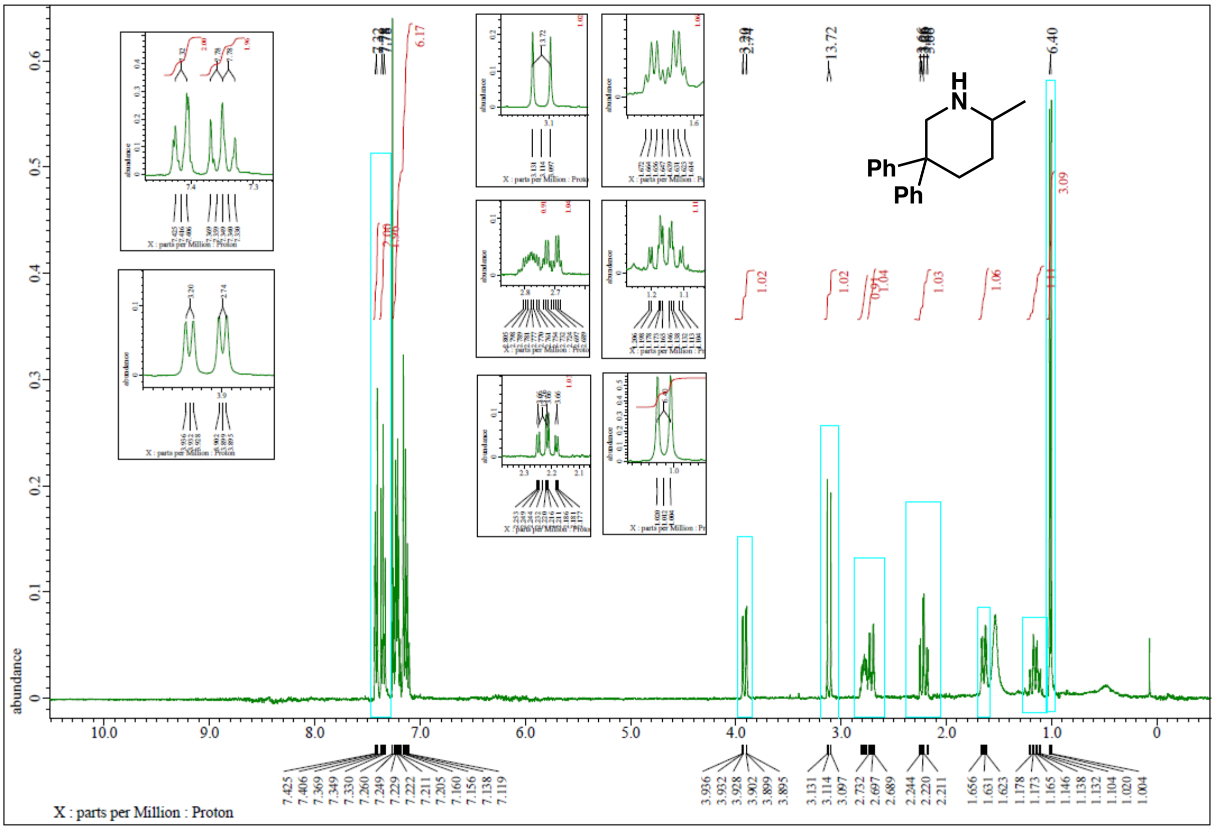

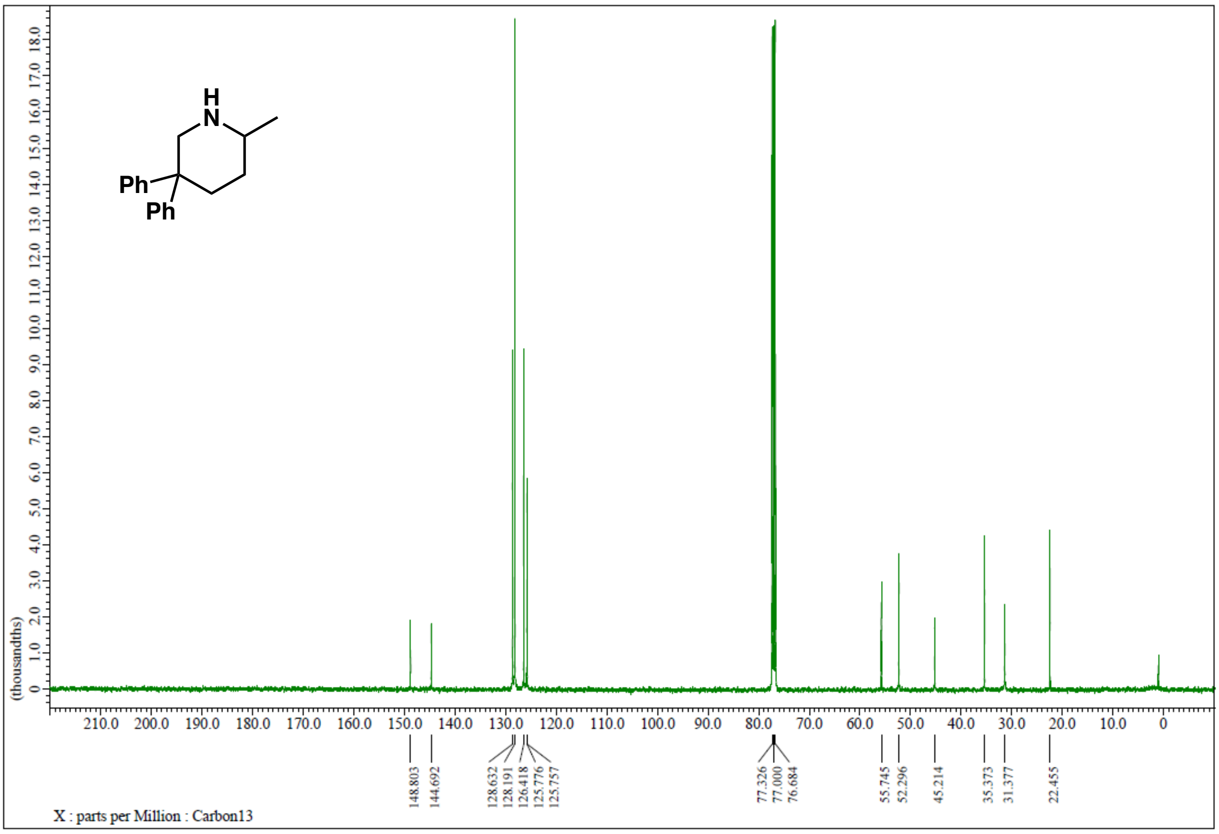

^{1}H NMR (400 MHz) and ^{13}C NMR (100 MHz) spectra of **2j** ($CDCl_3$)

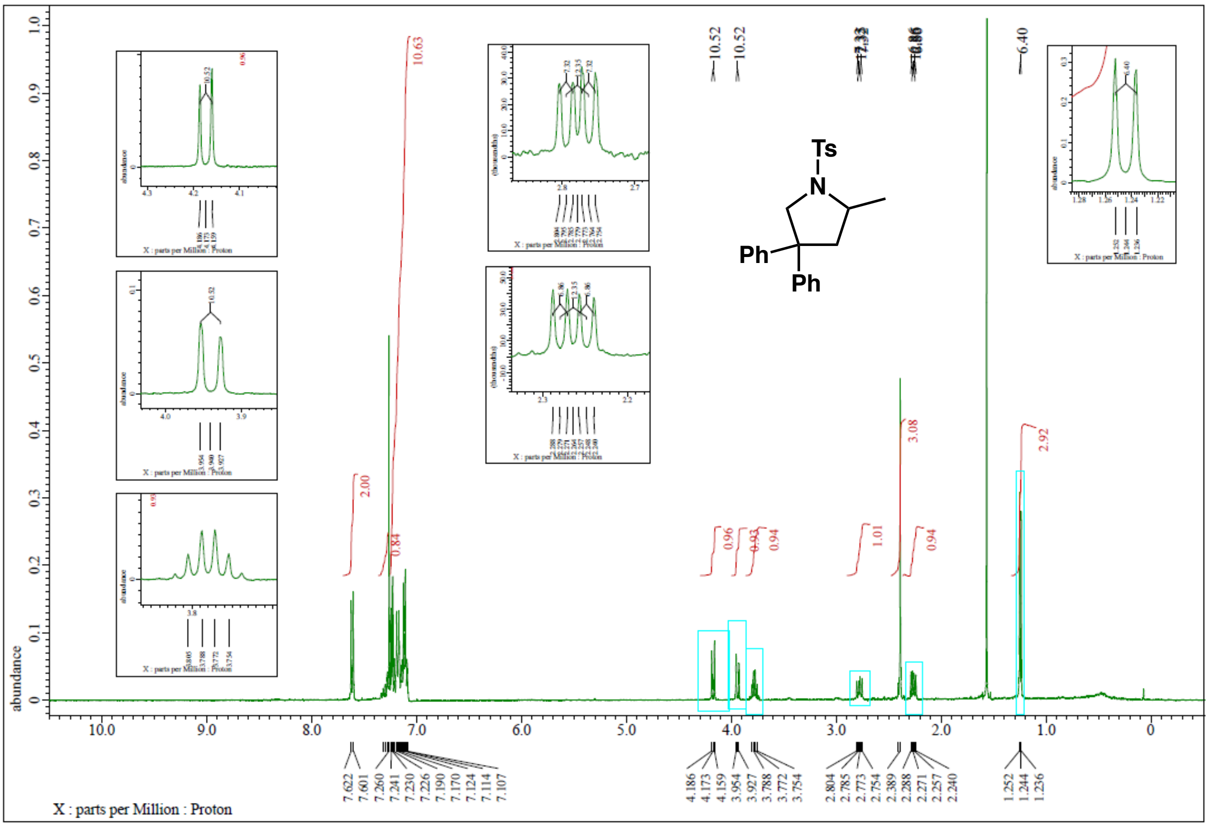

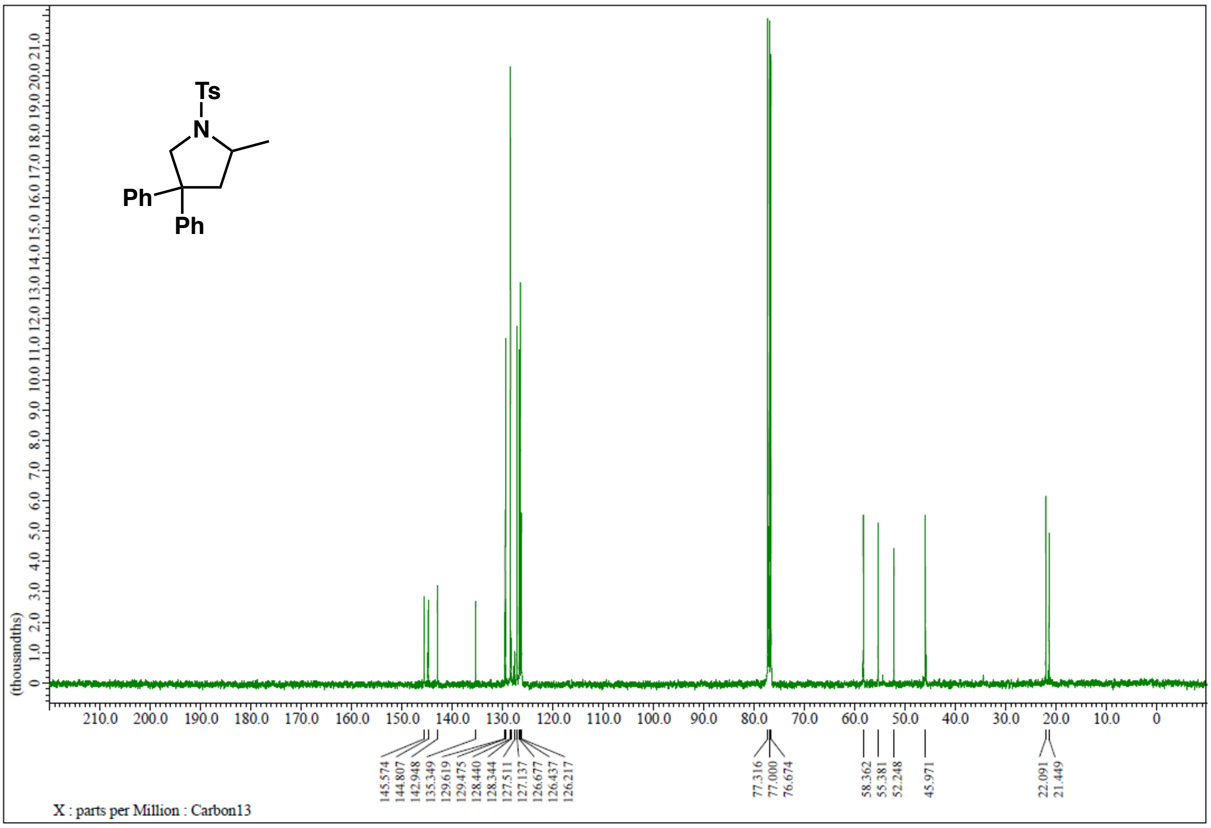

Supplement: Supplementary file 1 — Supplementary Information. [file 41598_2022_24955_MOESM1_ESM.pdf]
